# Rosmarinic Acid Attenuates Cadmium-Induced Nephrotoxicity via Inhibition of Oxidative Stress, Apoptosis, Inflammation and Fibrosis

**DOI:** 10.3390/ijms20082027

**Published:** 2019-04-24

**Authors:** Swarnalata Joardar, Saikat Dewanjee, Shovonlal Bhowmick, Tarun K. Dua, Sonjit Das, Achintya Saha, Vincenzo De Feo

**Affiliations:** 1 Advanced Pharmacognosy Research Laboratory, Department of Pharmaceutical Technology, Jadavpur University, Kolkata 700032, India; swarnalatajoardar@yahoo.in (S.J.); tarunkduaju@gmail.com (T.K.D.); dsonjit@gmail.com (S.D.); 2 Department of Chemical Technology, University of Calcutta, Kolkata 700009, India; sovonlal@gmail.com (S.B.); achintya_saha@yahoo.com (A.S.); 3 Department of Pharmacy, University of Salerno, 84084 Fisciano, Italy

**Keywords:** antioxidant, apoptosis, cadmium, oxidative stress, rosmarinic acid

## Abstract

The present investigation was executed to reveal the protective mechanism of rosmarinic acid (RA) against cadmium (Cd)-induced nephrotoxicity. RA exhibited a concentration-dependent anti-apoptotic effect against CdCl_2_ in isolated mouse proximal tubular epithelial cells. Cd treatment significantly (*p* < 0.01) imparted oxidative stress to the renal cells via excessive ROS production, triggering NO level, NADPH oxidase activation, and impairment of cellular redox defense system. Cd-mediated oxidative stress significantly (*p* < 0.01) endorsed apoptosis to the murine kidney cells by triggering NF-κB/PKC-δ/TNFR2 activation. In addition, CdCl_2_ induced renal fibrosis by triggering TGF-β1/SMAD3/α-SMA/collagen signaling within renal cells. On the other hand, RA significantly (*p* < 0.05–0.01) attenuated Cd-provoked oxidative stress and associated pathological signal transduction in murine renal cells. RA treatment also could significantly (*p* < 0.05–0.01) reciprocate Cd-mediated pathological changes in blood and urine parameters in mice. In addition, histological data supported the pharmacological findings. In silico chemometric analyses predicted the possible interactions between RA and different signal proteins and anticipated drug-likeness characteristics of RA. Hence, RA can potentially be applied as a therapeutic agent to treat Cd-mediated nephrotoxicity in future.

## 1. Introduction

Cadmium (Cd) is a heavy metal, which is released to the biosphere from both natural and anthropogenic sources [1]. It is considered to be a hazardous pollutant affecting almost all the vital organs in the body [1]. Main anthropogenic sources of Cd comprise nonferrous metal production, combustion of fossil fuel, fertilizers manufacturing, and incineration/burning of industrial wastes [2]. A dramatic increase in the release of bivalent Cd compounds to the biosphere coupled with the absence of an efficient recycling process resulted in a significant Cd burden in the earth crust, and Cd poisoning was revealed to be a serious health concern in many parts around the globe [3]. Cd can enter into human body via inhalation, ingestion, and dermal contact [4]. A significantly high extent of absorption coupled with a low rate of excretion corresponds to a high biological half-life (>20 years), which subsequently results in an enhancement of the Cd burden in various organs. However, it gets mostly accumulated within the kidneys and impairs its functions [4]. With chronic exposure, Cd accumulates in the epithelial cells of the renal proximal tubule, which hampers the function of tubular reabsorption, resulting in polyuria and proteinuria. The general pathological effects of Cd on renal functions were revealed; however, the specific molecular mechanisms are yet to be fully understood [5]. Studies over past few decades revealed that Cd-provoked oxidative stress is one of the most accepted mechanisms of Cd-mediated toxic manifestations in the kidneys and other organs [1,6,7]. Cd is a redox-stable metal, which promotes radical production via some indirect mechanisms [8]. One of the proposed mechanisms is the disruption in the cellular antioxidants, which further promotes enhanced production and accumulation of oxidative free radicals within the cells [9]. In addition, Cd can enhance the cellular levels of free redox-active metals via generation of free iron by replacing iron from various proteins, and these redox-active metals can promote free radical production via the Fenton reaction [6]. Cd-mediated oxidative stress within the renal cells is proposed to trigger the redox-specific pathological signal transductions and induce redox damage and apoptosis to the renal cells. Some of the key signaling pathways involved in the renal injury reported to be triggered by Cd include protein kinase C (PKC), nitric oxide (NO), nuclear factor-κB (NF-κB), tumor necrosis factor receptor (TNFR), transforming growth factor beta 1 (TGF-β1), α smooth muscle actin (α-SMA), E-cadherin, and collagen [5,10,11]. Therefore, it could be said that Cd induces nephrotoxicity via complex intracellular signaling pathways principally governed by oxidative stress. Amongst the therapeutic strategies, it is advocated that a reduction of Cd bioaccumulation employing metal-chelating agents and a reduction of Cd-provoked oxidative stress using natural and synthetic antioxidants remain the two major approaches to attenuate Cd-toxicity. As of yet, no such metal chelating agent is proven to be successful against Cd toxicity. In addition, metal-chelating agents were reported to possess several untoward effects, such as redistribution/translocation of metals and serious toxic manifestations; for example, 2,3-dimercaptopropanol induces cardiotoxicity and hypersensitivity; d-penicillamine imparts hematotoxicity; succimer causes hypersensitivity; CaNa_2_EDTA imparts nephrotoxicity and zinc dieresis, etc. [12]. On the other hand, various naturally occurring phenolic antioxidants were reported to possess a simultaneous bivalent metal-chelating property [13]. Therefore, it would be reasonable to pay them special attention so as to develop a pharmacotherapeutic agent from the naturally occurring phenolic antioxidants to be effective in attenuating Cd-mediated nephrotoxicity.

**Figure 1:**
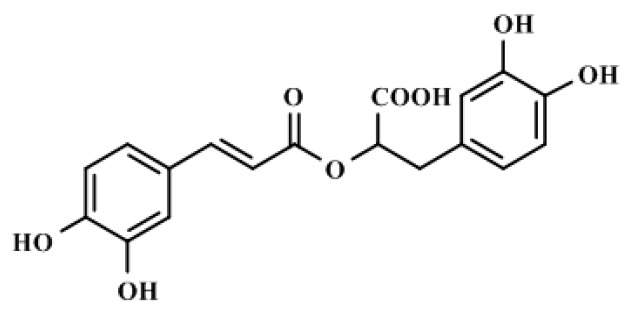
Chemical structure of RA or (2*R*)-3-(3,4-dihydroxyphenyl)-2-{[(2*E*)-3-(3,4-dihydroxyphenyl)-2-propenoyl]oxy}propanoic acid (IUPAC).

Rosmarinic acid (RA; Figure 1), a naturally occurring polyphenolic nutraceutical, is an ester of caffeic acid and 3,4-dihydroxyphenyllactic acid belonging to the class of phenylpropanoids [14]. RA is an active constituent of *Rosmarinus officinalis*. This hydrophilic compound is found within a wide variety of plant species [15]. RA was reported to possess antioxidant, anti-inflammatory, antimicrobial, hepatoprotective, cardio-protective, anti-mutagenic, anti-cancer, antidepressant, antiangiogenic, and antiallergenic activities [15]. In addition, RA was reported to possess a metal-chelating property [13]. Considering the antioxidant and metal-chelating effects of RA, the present investigation was undertaken to evaluate the possible therapeutic potential of RA against Cd-induced nephrotoxicity employing appropriate in vitro and in vivo preclinical assays. Special attention was given to explore possible molecular mechanism(s) behind the antinephrotoxic effect of RA. Finally, in silico prediction of probable absorption, distribution, metabolism, and excretion (ADME) characteristics and molecular docking studies were undertaken to predict the probable drug-likeness and molecular interaction patterns of RA, respectively.

## 2. Results

### 2.1. Effect of RA on CdCl_2_-Mediated Toxicity in Vitro

#### 2.1.1. Dose-Dependent Cytotoxic Effect of CdCl_2_

**Figure 2:**
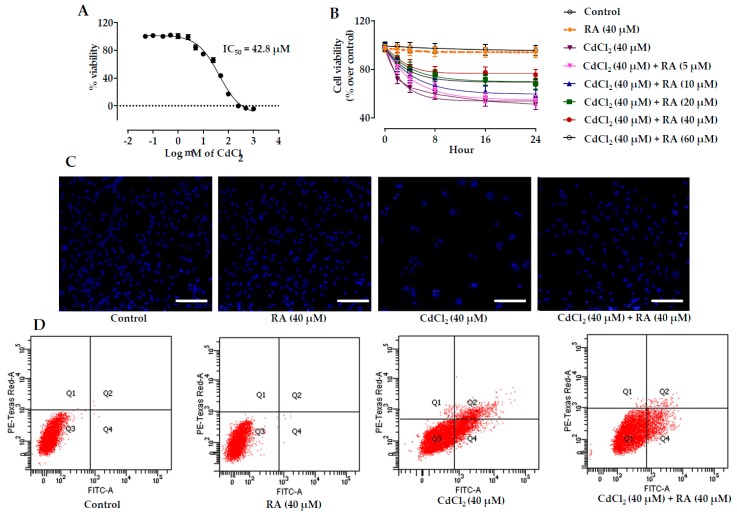
The effects on the cell viability, Hoechst nuclear staining, and flow cytometric analysis in the absence (CdCl_2_) and in the presence of RA (CdCl_2_ + RA) in vitro on isolated mouse proximal tubular epithelial cells. (**A**) Effect of CdCl_2_ at different concentrations in the cell viability on murine proximal tubular epithelial cells. (**B**) Effect on the cell viability in the absence (CdCl_2_) and presence of RA (CdCl_2_ + RA) on murine renal tubular cells. (**C**) Effect on Hoechst staining in the absence (CdCl_2_) and presence of RA (CdCl_2_ + RA) on murine renal tubular cells. Scale = 20 µm. (**D**) Percentage of apoptotic and necrotic cells in the absence (CdCl_2_) and presence of RA (CdCl_2_ + RA) on murine renal tubular cells analyzed by flow cytometric assay. Data are represented as means ± SD, *n* = 3 (number of plates).

The cytotoxic effect of CdCl_2_ was evaluated by incubating mouse proximal tubular epithelial cells with different concentrations of CdCl_2_ for 24 h. CdCl_2_-exposed renal cells exhibited loss of cell viability in a concentration-dependent manner (Figure 2A). The IC_50_ value was found to be 42.8 μM and subsequent in vitro studies were performed using CdCl_2_ (40 μM) as a toxic control.

#### 2.1.2. Cytoprotective Effects of RA

Mouse proximal tubular epithelial cells incubated with CdCl_2_ (40 μM) exhibited gradual reduction in the viability of cells up to 24 h (Figure 2B). On the other hand, simultaneous incubation of cells with RA (5–60 μM) 1 h prior to CdCl_2_ (40 μM) treatment significantly reciprocated CdCl_2_-induced cytotoxicity to the mouse proximal tubular epithelial cells up to 24 h (Figure 2B). However, the best cytoprotective effect of RA was found at the concentration of 40 µM, which was optimized for subsequent in vitro experiments. The cells incubated with RA (40 µM) did not show any significant changes in the cell viability when compared with untreated murine proximal tubular epithelial cells up to 24 h (Figure 2B).

#### 2.1.3. Effects on Hoechst Staining

Hoechst nuclear staining was performed to visualize the cytoprotective effect of RA (40 µM) (Figure 2C). After 24 h, the set exposed to CdCl_2_ (40 μM) revealed a significant reduction in the number of visible nuclei in association with the induction of apoptosis when compared with the untreated set. On the other hand, incubation of cells with RA (40 μM) 1 h prior to CdCl_2_ (40 μM) treatment significantly reciprocated nuclear count, and restored nuclear morphology to near-normal status. The cells incubated with RA (40 µM) for 24 h did not show any significant change in the nuclear count or nuclear morphology when compared with untreated murine proximal tubular epithelial cells.

#### 2.1.4. Flow Cytometric Analysis

CdCl_2_-treated (40 μM) murine renal cells exhibited low PI staining (~0.1%) with very high annexin V-FITC binding (~46.3%), indicating induction of apoptosis to the murine proximal tubular epithelial cells when compared with untreated cells, which showed very little annexin V-FITC binding (~0.04%) and PI staining (~0.1%) (Figure 2D). On the other hand, incubation of mouse proximal tubular epithelial cells with RA (40 µM) 1 h prior to CdCl_2_ (40 μM) attenuated CdCl_2_-induced apoptosis, apparent from the reduction in apoptotic cell counts (Figure 2D). On the other hand, the cells incubated with RA (40 µM) alone for 24 h did not show any significant change in scatter plots of PI fluorescence vs. FITC fluorescence when compared with untreated murine renal cells (Figure 2D).

#### 2.1.5. Effects on Redox Status In Vitro

**Figure 3:**
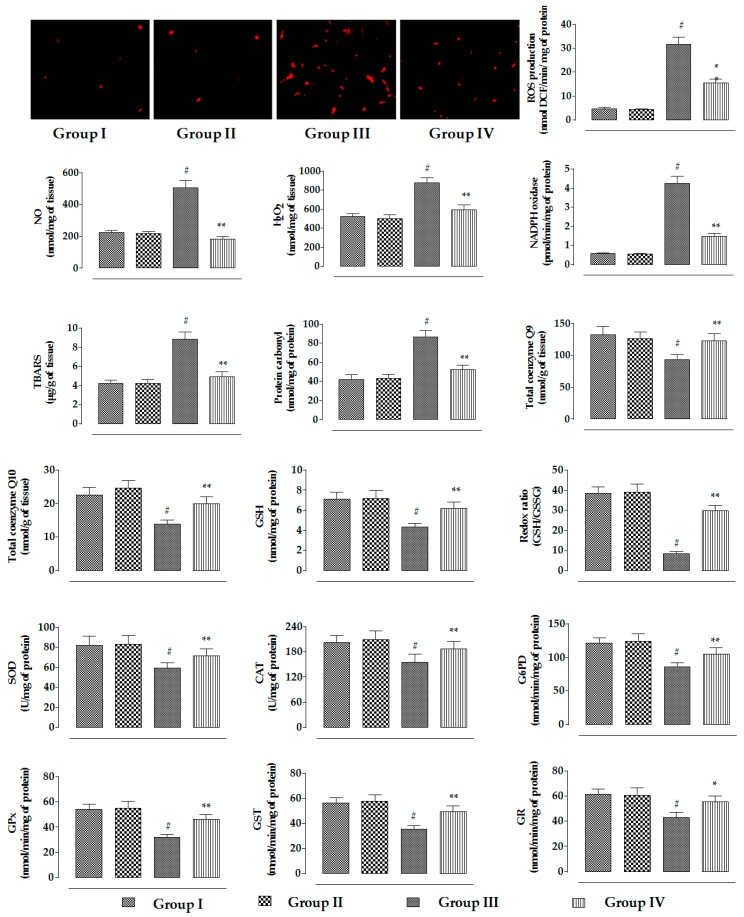
The effects on the ROS accumulation, NO production, H_2_O_2_ level, NADPH oxidase activity, extent of lipid peroxidation, extent of protein carbonylation, co-enzyme Q9 level, co-enzyme Q10 level, and the levels of endogenous redox markers in the absence (CdCl_2_) and presence of RA (CdCl_2_ + RA) in vitro on mouse proximal tubular epithelial cells. Data are represented as means ± SD, *n* = 3 (number of plates). ^#^ Values significantly (*p* < 0.01) differ from normal control. * Values significantly (*p* < 0.05) differ from CdCl_2_ control. ** Values significantly (*p* < 0.01) differ from CdCl_2_ control. SOD unit, “U”, is defined as inhibition (μ moles) of NBT reduction/min. CAT unit, “U”, is defined as H_2_O_2_ consumption/min. Group I: untreated, Group II: RA (40 µM), Group III: CdCl_2_ (40 µM), and Group IV: CdCl_2_ (40 µM) + RA (40 µM).

CdCl_2_ (40 μM) treatment to murine renal cells caused a significant (*p* < 0.01) increase in the ROS production supported by the augmentation in DCF fluorescence as compared to untreated cells (Figure 3). In addition, CdCl_2_ (40 μM) treatment significantly (*p* < 0.01) enhanced the levels of NO, H_2_O_2_, and NADPH oxidase in murine proximal tubular epithelial cells (Figure 3). The CdCl_2_-mediated oxidative stress consequently endorsed lipid peroxidation and protein carbonylation. The degree of lipid peroxidation was quantified by measuring the level of thiobarbituric acid-reactive substances (TBARS), which is a byproduct of lipid peroxidation. Significant (*p* < 0.01) increment in the levels of TBARS and carbonylated proteins was observed in CdCl_2_-treated (40 μM) murine renal cells (Figure 3). On the other hand, RA (40 µM) treatment 1 h prior to CdCl_2_ (40 μM) significantly (*p* < 0.01) attenuated the CdCl_2_-induced increase in ROS production, NO level, H_2_O_2_ content, NADPH oxidase level, lipid peroxidation, and protein carbonylation in mouse proximal tubular epithelial cells (Figure 3). In this study, CdCl_2_ (40 μM) treatment significantly (*p* < 0.01) reduced the levels of co-enzymes Q9 and Q10 in mouse renal cells (Figure 3). On the other hand, RA (40 µM) treatment 1 h prior to CdCl_2_ (40 μM) significantly (*p* < 0.01) reciprocated co-enzymes Q9 and Q10 levels in mouse renal cells (Figure 3). CdCl_2_ (40 μM) treatment further induced oxidative stress to the murine renal cells via significant (*p* < 0.01) depletion in the levels of GSH and cellular antioxidant enzymes, such as SOD, CAT, GPx, GST, GR, and G6PD. On the other hand, incubation of mouse proximal tubular epithelial cells with RA (40 µM) 1 h prior to CdCl_2_ (40 μM) significantly (*p* < 0.05–0.01) reciprocated GSH and endogenous antioxidant enzymes. On the other hand, the cells incubated with RA (40 µM) alone for 24 h did not show any significant change in any of the aforementioned redox parameters (Figure 3).

#### 2.1.6. Effects on Signal Proteins In Vitro

**Figure 4:**
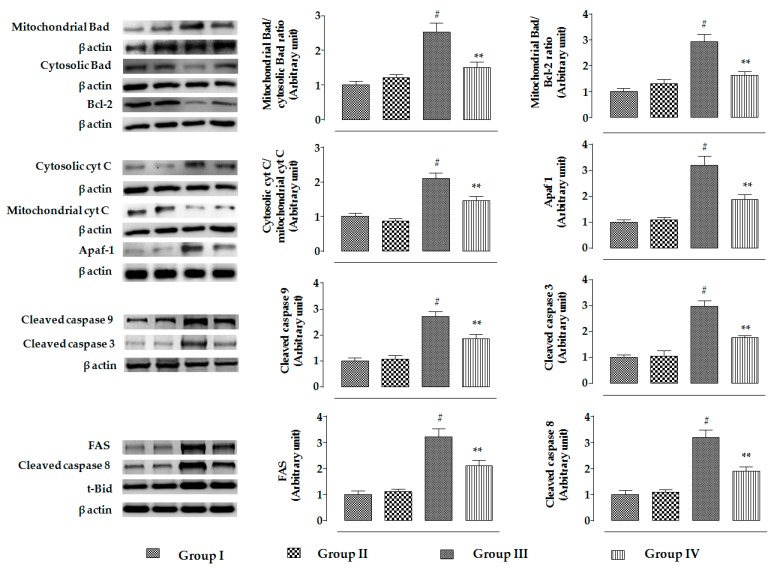
The effects on apoptotic signaling in the absence (CdCl_2_) and presence of RA (CdCl_2_ + RA) in vitro on mouse proximal tubular epithelial cells. The relative band intensities were assessed, and the intensity of the normal control band was assigned as 1. β-Actin served as the loading control. Data are represented as means ± SD, *n* = 3 (number of plates). ^#^ Values significantly (*p* < 0.01) differ from normal control. ** Values significantly (*p* < 0.01) differ from CdCl_2_ control. Group I: untreated, Group II: RA (40 µM), Group III: CdCl_2_ (40 µM), and Group IV: CdCl_2_ (40 µM) + RA (40 µM).

The changes in the signaling events in mouse proximal tubular epithelial cells were assessed by immunoblotting. In this study, incubation of mouse proximal tubular epithelial cells with CdCl_2_ (40 μM) promoted the mitochondrial translocation of pro-apoptotic Bad protein from cytosol, resulting in a significantly (*p* < 0.01) high ratio of mitochondrial Bad to cytosolic Bad (Figure 4). CdCl_2_ (40 μM) further caused downregulation in the expression of anti-apoptotic proteins, such as Bcl-2, resulting in a significantly (*p* < 0.01) high ratio of mitochondrial Bad to cellular Bcl-2 (Figure 4). On the other hand, incubation of mouse proximal tubular epithelial cells with RA (40 µM) 1 h prior to CdCl_2_ (40 μM) treatment significantly (*p* < 0.01) reciprocated activation of pro-apoptotic Bad and downregulation of Bcl-2 to near-normal status mouse renal cells. CdCl_2_ (40 μM) treatment further caused the release of cytochrome C into the cytosol from mitochondria, resulting in a significantly (*p* < 0.01) high ratio of cytosolic cytochrome C to mitochondrial cytochrome C (Figure 4). On the other hand, incubation of mouse proximal tubular epithelial cells with RA (40 µM) 1 h prior to CdCl_2_ (40 μM) treatment significantly (*p* < 0.01) inhibited cytochrome C release into the cytosol (Figure 4). Activation of pro-apoptotic protein, impairment of anti-apoptotic protein, and release of cytochrome C into the cytosol sequentially promoted intrinsic apoptotic signaling, evidenced from the upregulation (*p* < 0.01) of Apaf-1, and cleavages (*p* < 0.01) of caspases 9 and 3 in CdCl_2_-exposed (40 μM) mouse proximal tubular epithelial cells (Figure 4). On the other hand, incubation of mouse renal cells with RA (40 µM) 1 h prior to CdCl_2_ (40 μM) treatment significantly (*p* < 0.01) attenuated the activation of Apaf-1, and cleavages (*p* < 0.01) of caspases 9 and 3 (Figure 4). Significant upregulation in the expressions of proteins involved in death receptor-mediated apoptosis, such as FAS, t-Bid, and cleaved caspase 8 was observed in CdCl_2_-exposed (40 μM) mouse proximal tubular epithelial cells (Figure 4). On the other hand, incubation of mouse proximal tubular epithelial cells with RA (40 µM) 1 h prior to CdCl_2_ (40 μM) treatment significantly (*p* < 0.01) attenuated the activation of FAS and caspase 8. However, RA (40 µM) could not significantly reinstate t-Bid expression (data were not included).

**Figure 5:**
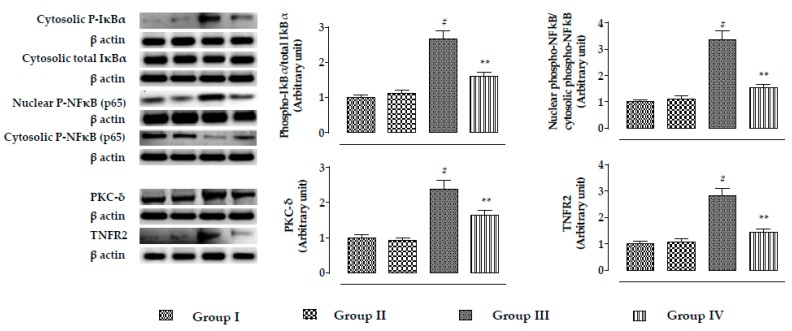
The effects on NF-κB, PKC-δ, and TNFR2 expressions in the absence (CdCl_2_) and presence of RA (CdCl_2_ + RA) in vitro on mouse proximal tubular epithelial cells. The relative band intensities were assessed, and the intensity of the normal control band was assigned as 1. β-Actin served as the loading control. Data are represented as means ± SD, *n* = 3 (number of plates). ^#^ Values significantly (*p* < 0.01) differ from normal control.. ** Values significantly (*p* < 0.01) differ from CdCl_2_ control. Group I: untreated, Group II: RA (40 µM), Group III: CdCl_2_ (40 µM), and Group IV: CdCl_2_ (40 µM) + RA (40 µM).

The expressions of NF-κB, PKC-δ, and TNFR2 are shown in Figure 5. In this study, incubation of mouse proximal tubular epithelial cells with CdCl_2_ (40 μM) significantly (*p* < 0.01) promoted phosphorylation of IκBα in the cytosol, which simultaneously activated NF-κB signaling via its phosphorylation followed by nuclear translocation. A significantly (*p* < 0.01) high ratio of nuclear phospho-NF-κB to cytosolic phospho-NF-κB was recorded in CdCl_2_-exposed (40 μM) mouse renal cells. In addition, CdCl_2_ (40 μM) caused significant (*p* < 0.01) upregulation in the expressions of PKC-δ and TNFR2 in mouse renal cells. On the other hand, incubation of mouse proximal tubular epithelial cells with RA (40 µM) 1 h prior to CdCl_2_ (40 μM) treatment significantly (*p* < 0.01) reciprocated NF-κB, PKC-δ, and TNFR2 activation in mouse renal cells. 

**Figure 6:**
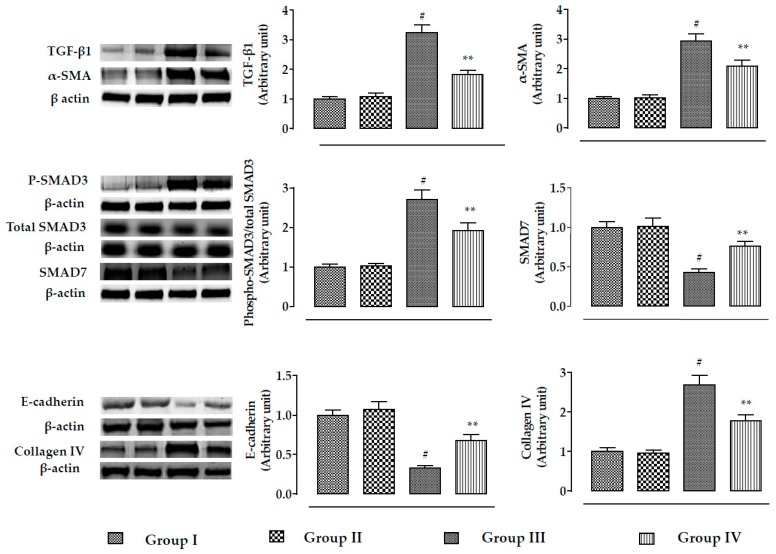
The effects on TGF-β1, α-SMA, SMAD3 SMAD7, E-cadherin, and collagen-IV expressions in the absence (CdCl_2_) and presence of RA (CdCl_2_ + RA) in vitro on mouse proximal tubular epithelial cells. The relative band intensities were assessed, and the intensity of the normal control band was assigned as 1. β-Actin served as the loading control. Data are represented as means ± SD, *n* = 3 (number of plates). ^#^ Values significantly (*p* < 0.01) differ from normal control. *Values significantly (*p* < 0.05) differ from CdCl_2_ control. ** Values significantly (*p* < 0.01) differ from CdCl_2_ control. Group I: untreated, Group II: RA (40 µM), Group III: CdCl_2_ (40 µM), and Group IV: CdCl_2_ (40 µM) + RA (40 µM).

The effect of the TGF-β/SMAD/collagen VI signaling pathway is depicted in Figure 6. In this study, incubation of mouse renal cells with CdCl_2_ (40 μM) significantly (*p* < 0.01) activated TGF-β1, α-SMA, and phospho-SMAD3 expressions, while significant (*p* < 0.01) impediment of SMAD7 expression was observed. On the other hand, incubation of mouse proximal tubular epithelial cells with RA (40 µM) 1 h prior to CdCl_2_ (40 μM) treatment significantly (*p* < 0.05–0.01) reciprocated TGF-β1, α-SMA, SMAD3, and SMAD7 signaling. In addition, CdCl_2_ (40 μM) significantly (*p* < 0.01) activated collagen IV expression and downregulated the expression of E-cadherin in mouse proximal tubular epithelial cells (Figure 6). However, incubation of mouse renal cells with RA (40 µM) 1 h prior to CdCl_2_ (40 μM) treatment significantly (*p* < 0.01) reciprocated E-cadherin and collagen IV expressions (Figure 6).

On the other hand, the cells incubated with RA (40 µM) alone for 24 h did not show any significant change in either of aforementioned signal transduction (Figure 4, Figure 5 and Figure 6).

### 2.2. Effect on CdCl_2_-Mediated Nephrotoxicity In Vivo

#### 2.2.1. Effect on Serum Biochemical Parameters

The effects of RA on blood parameters are shown in Table 1. CdCl_2_ (4 mg/kg) treatment significantly (*p* < 0.01) increased total cholesterol and triglyceride levels in the sera of experimental mice, while a significant decrease in serum HDL cholesterol level was observed (*p* < 0.01) in CdCl_2_-treated mice. Furthermore, a significant (*p* < 0.01) increment of membrane-bound enzymes, such as LDH and CK was recorded in CdCl_2_-treated mice. In addition, CdCl_2_ (4 mg/kg) treatment caused significant (*p* < 0.01) increase in the levels of urea, uric acid, and creatinine in the sera of experimental mice. On the other hand, RA (50 mg/kg) significantly (*p* < 0.05–0.01) reciprocated CdCl_2_-mediated pathological changes in serum biochemical parameters. However, RA (50 mg/kg) alone could not impart any significant change in the aforementioned biochemical parameters of sera of experimental mice when compared to that of untreated group.

#### 2.2.2. Effect on Kidney Mass, Cd Accumulation in Kidneys, and Urine Parameters

In this study, CdCl_2_ (4 mg/kg) treatment for two weeks significantly increased kidney mass (*p* < 0.05), kidney mass/body mass (*p* < 0.01), and Cd bio-accumulation in the kidneys when compared to the untreated control (Table 2). On the other hand, RA (50 mg/kg) significantly reciprocated CdCl_2_-mediated increment in the kidney mass (*p* < 0.05), kidney mass/body mass (*p* < 0.05), and renal Cd bio-accumulation (*p* < 0.05) when compared to the CdCl_2_ control group (Table 2). In the study of urinary parameters, CdCl_2_ (4 mg/kg) treatment for two weeks significantly (*p* < 0.01) increased urinary creatinine, urinary albumin, and urinary nitrate/nitrite levels when compared to the untreated mice (Table 2). On the other hand, RA (50 mg/kg) significantly reciprocated the CdCl_2_-mediated increase in creatinine, albumin, and nitrate/nitrite levels in the urine of experimental mice as compared with the CdCl_2_ control group (Table 2). However, RA (50 mg/kg) alone could not impart any significant change in the kidney mass, Cd accumulation, and urinary biochemical profile of experimental mice when compared to the untreated group (Table 2).

#### 2.2.3. Effects on Inflammatory Mediators

**Figure 7:**
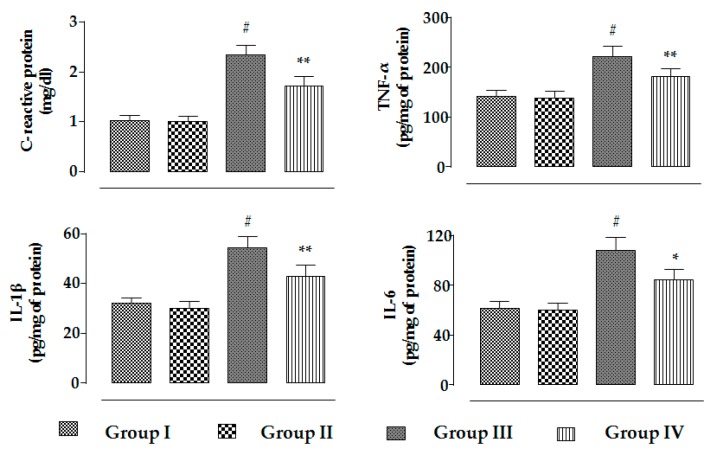
The effects on serum inflammatory markers, i.e., C-reactive proteins, THF-α, IL-1β, and IL-6, in the absence (CdCl_2_) and presence of RA (CdCl_2_ + RA) in vivo on mouse kidneys. Data are represented as means ± SD, *n* = 6 (number of plates). ^#^ Values significantly (*p* < 0.01) differ from normal control. * Values significantly (*p* < 0.05) differ from CdCl_2_ control. ** Values significantly (*p* < 0.01) differ from CdCl_2_ control.

In this study, CdCl_2_ (4 mg/kg) treatment for two weeks significantly (*p* < 0.01) enhanced the levels of C-reactive proteins, TNF-α, IL-1β, and IL-6 in the sera of experimental mice (Figure 7). On the other hand, RA (50 mg/kg) significantly reduced CdCl_2_-mediated enhancement in the C-reactive protein (*p* < 0.01), TNF-α (*p* < 0.01), IL-1β (*p* < 0.01), and IL-6 (*p* < 0.05) levels in the sera of mice (Figure 7). However, RA (50 mg/kg) alone could not impart any change in the level of inflammatory markers in the sera of experimental mice when compared to the untreated group (Figure 7).

#### 2.2.4. Effects on Redox Status In Vivo

**Figure 8:**
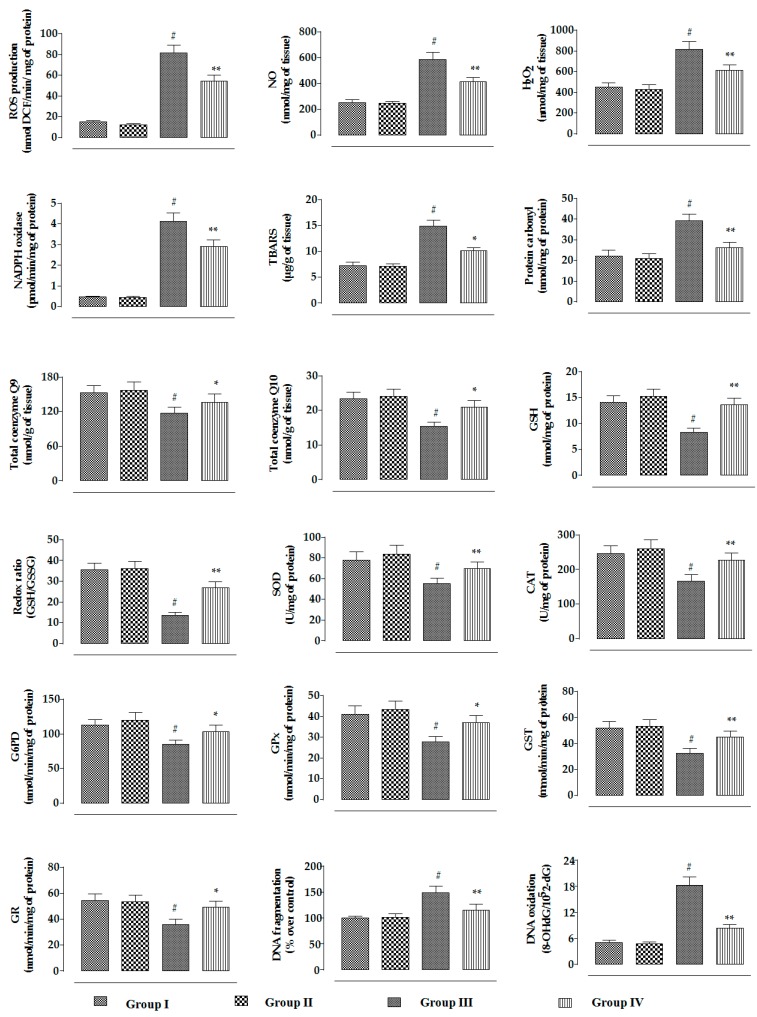
The effects on the ROS accumulation, NO production, H_2_O_2_ level, NADPH oxidase activity, extent of lipid peroxidation, extent of protein carbonylation, co-enzyme Q9 level, co-enzyme Q10 level, the levels of endogenous redox markers, degree of DNA fragmentation, and extent of DNA oxidation in the absence (CdCl_2_) and presence of RA (CdCl_2_ + RA) in vivo on mouse kidneys. Data are represented as mean ± SD, *n* = 6 (number of plates). ^#^ Values significantly (*p* < 0.01) differ from normal control. * Values significantly (*p* < 0.05) differ from CdCl_2_ control. ** Values significantly (*p* < 0.01) differ from CdCl_2_ control. SOD unit, “U”, is defined as inhibition (μ moles) of NBT reduction/min. CAT unit, “U”, is defined as H_2_O_2_ consumption/min.

In this study, CdCl_2_ (4 mg/kg) treatment for two weeks significantly (*p* < 0.01) promoted NADPH oxidase level in renal tissue of mice, resulting in a significant (*p* < 0.01) increase in the ROS, NO, and H_2_O_2_ contents in the kidneys of experimental mice (Figure 8). On the other hand, RA (50 mg/kg) significantly (*p* < 0.01) reduced CdCl_2_-mediated increase in the NADPH oxidase, ROS, NO, and H_2_O_2_ levels in the kidneys of experimental mice (Figure 8). CdCl_2_-mediated generation of excessive oxidative radicals and H_2_O_2_ caused significant (*p* < 0.01) oxidative damage to the cellular lipids and proteins via peroxidation and carbonylation, respectively (Figure 8). CdCl_2_ (4 mg/kg) treatment further promoted oxidative stress evidenced from the reduced levels of endogenous antioxidant enzymes in the kidneys of experimental mice (Figure 8). Significant (*p* < 0.01) decreases in GSH level and redox ratio (GSH/GSSG) were found in the renal tissue of CdCl_2_-treated mice (Figure 8). In this study, CdCl_2_ (4 mg/kg) treatment for two weeks significantly (*p* < 0.01) reduced the levels of co-enzymes Q9 and Q10 in the kidneys of experimental mice (Figure 8). CdCl_2_-mediated (4 mg/kg) oxidative stress induced DNA fragmentation and DNA oxidation in the kidneys of experimental mice, evidenced from significant (*p* < 0.01) increases in the percentage of fragmented DNA and 8-OHdG/10^5^ × 2-dG over normal control group. On the other hand, RA (50 mg/kg) significantly (*p* < 0.05–0.01) reciprocated CdCl_2_-mediated enhancement of oxidative stress and associated oxidative damage in the kidneys of mice (Figure 8). RA (50 mg/kg) alone could not impart any change in radical generation, redox stress, and associated oxidative damages in the kidneys of experimental mice when compared to the untreated group (Figure 8).

#### 2.2.5. Effects on Signal Proteins In Vivo

**Figure 9:**
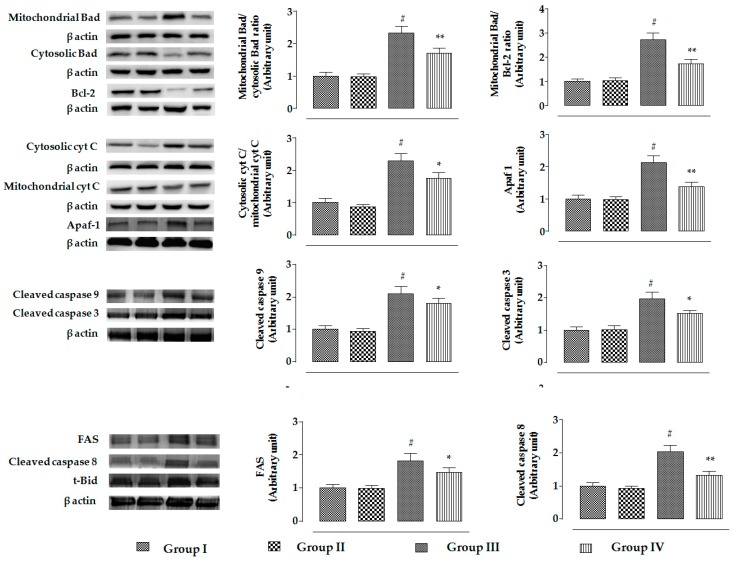
The effects on intrinsic and extrinsic apoptotic signaling in the absence (CdCl_2_) and presence of RA (CdCl_2_ + RA) in vivo on mouse kidneys. The relative band intensities were assessed, and the intensity of the normal control band was assigned as 1. β-Actin served as the loading control. Data are represented as means ± SD, *n* = 6 (number of plates). ^#^ Values significantly (*p* < 0.01) differ from normal control. * Values significantly (*p* < 0.05) differ from CdCl_2_ control. ** Values significantly (*p* < 0.01) differ from CdCl_2_ control.

The changes in the signaling events in the specific cellular components of mouse kidneys were assessed by Western blotting. In this study, CdCl_2_ (4 mg/kg) treatment for two weeks significantly promoted the mitochondrial translocation of pro-apoptotic Bad protein from cytosol in the kidneys of mice, resulting in a significantly (*p* < 0.01) high ratio of mitochondrial Bad to cytosolic Bad (Figure 9). Simultaneously, CdCl_2_ (4 mg/kg) treatment caused significant downregulation of anti-apoptotic Bcl-2 protein in the kidneys of mice, resulting in a significantly (*p* < 0.01) high ratio of mitochondrial Bad to Bcl-2 (Figure 9). On the other hand, RA (50 mg/kg) treatment significantly (*p* < 0.01) reciprocated CdCl_2_-mediated activation of pro-apoptotic Bad and downregulation of Bcl-2 to near-normal status in the kidneys of experimental mice (Figure 9). CdCl_2_-mediated reciprocation of pro-apoptotic and anti-apoptotic factors further instigated the release of cytochrome C into cytosol from mitochondria, followed by activation of Apaf-1, and cleavages of caspase 9 and 3 in the murine kidneys. In this study, a significantly (*p* < 0.01) high ratio of cytosolic cytochrome C to mitochondrial cytochrome C coupled with enhanced expressions of Apaf-1, and cleaved caspases 9 and 3 was observed in the kidneys of CdCl_2_-treated (4 mg/kg) mice (Figure 9). On the other hand, RA (50 mg/kg) treatment significantly inhibited CdCl_2_-mediated cytochrome C release to the cytosol (*p* < 0.05), activation of Apaf-1 (*p* < 0.01), and cleavages of caspase 9 (*p* < 0.05) and 3 (*p* < 0.05) in the murine kidneys (Figure 9). Significant upregulation in the expressions of FAS, t-Bid, and cleaved caspase 8 were observed in the kidneys of CdCl_2_ (4 mg/kg)-treated mouse (Figure 9). On the other hand, RA (50 mg/kg) treatment significantly (*p* < 0.01) attenuated the activation of FAS (*p* < 0.05) and caspase 8 (*p* < 0.01); however, RA (50 mg/kg) could not significantly reinstate t-Bid expression (data were not included).

**Figure 10:**
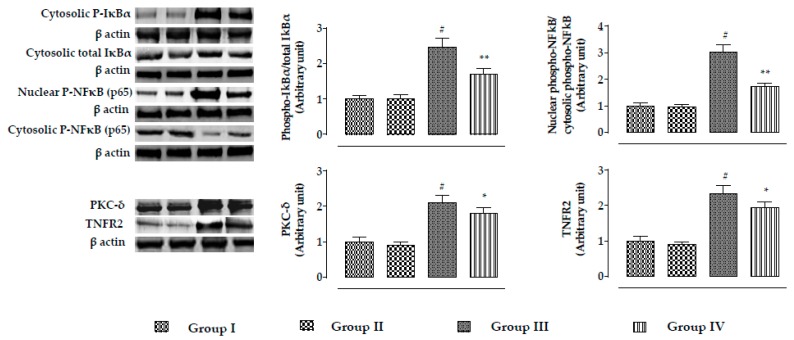
The effects on NF-κB, PKC-δ, and TNFR2 expressions in the absence (CdCl_2_) and presence of RA (CdCl_2_ + RA) in vivo on mouse kidneys. The relative band intensities were assessed, and the intensity of the normal control band was assigned as 1. β-Actin served as the loading control. Data are represented as means ± SD, *n* = 6 (number of plates). ^#^ Values significantly (*p* < 0.01) differ from normal control. * Values significantly (*p* < 0.05) differ from CdCl_2_ control. ** Values significantly (*p* < 0.01) differ from CdCl_2_ control.

In this study, CdCl_2_ (4 mg/kg) significantly (*p* < 0.01) promoted the phosphorylation of IκBα in the cytosol, which simultaneously activated NF-κB signaling via nuclear translocation of phospho- NF-κB in murine kidneys (Figure 10). A significantly (*p* < 0.01) high ratio of nuclear phospho-NF-κB to cytosolic phospho-NF-κB was found in the renal cells of CdCl_2_-treated (4 mg/kg) mice (Figure 10). CdCl_2_ (4 mg/kg) treatment for two weeks significantly promoted the expressions of PKC-δ and TNFR2 in the kidneys of mice (Figure 10). On the other hand, RA (50 mg/kg) treatment significantly attenuated NF-κB (*p* < 0.01), PKC-δ (*p* < 0.05), and TNFR2 (*p* < 0.05) activation in mouse kidneys (Figure 10).

**Figure 11:**
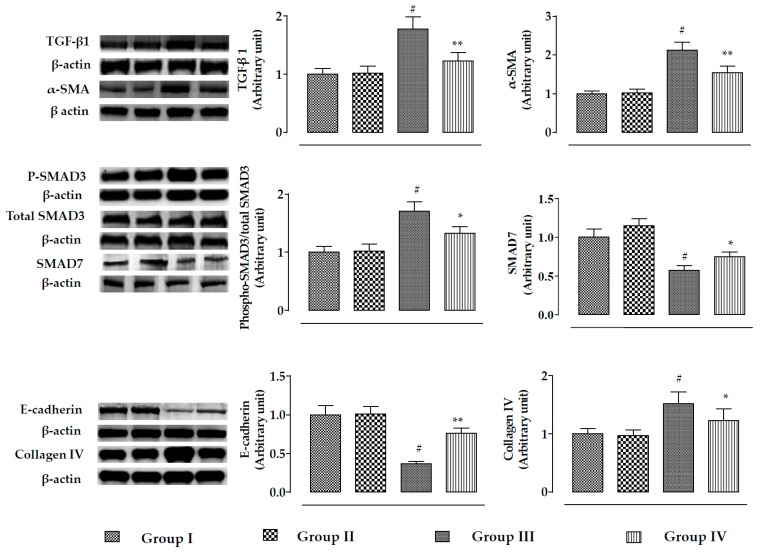
The effects on TGF-β1, α-SMA, SMAD3 SMAD7, E-cadherin, and collagen-IV expressions in the absence (CdCl_2_) and presence of RA (CdCl_2_ + RA) in vivo on mouse kidneys. The relative band intensities were assessed, and the intensity of the normal control band was assigned as 1. β-Actin served as the loading control. Data are represented as means ± SD, *n* = 6 (number of plates). ^#^ Values significantly (*p* < 0.01) differ from normal control. * Values significantly (*p* < 0.05) differ from CdCl_2_ control. ** Values significantly (*p* < 0.01) differ from CdCl_2_ control.

The effect of the TGF-β/SMAD/collagen VI signaling pathway is shown in Figure 11. In this study, CdCl_2_ (4 mg/kg) treatment for two weeks significantly activated (*p* < 0.01) TGF-β1, α-SMA, and phospho-SMAD3 expressions, while significant (*p* < 0.01) impairment of SMAD7 expression was observed. On the other hand, RA (50 mg/kg) treatment significantly reciprocated CdCl_2_-mediated alteration in TGF-β1 (*p* < 0.01), α-SMA (*p* < 0.01), phospho-SMAD3 (*p* < 0.05), and SMAD7 (*p* < 0.05) expressions. In addition, CdCl_2_ (4 mg/kg) treatment significantly (*p* < 0.01) promoted collagen IV expression and downregulated the expression of E-cadherin in the kidneys of experimental mice. On the other hand, RA (50 mg/kg) treatment significantly reversed CdCl_2_-mediated impairment in collagen IV and E-cadherin expressions. 

In this study, RA (50 mg/kg) alone could not impart any change in the expression of either signaling protein in the kidneys of experimental mice when compared to the untreated group.

#### 2.2.6. Effects on Histology of Kidneys

**Figure 12:**
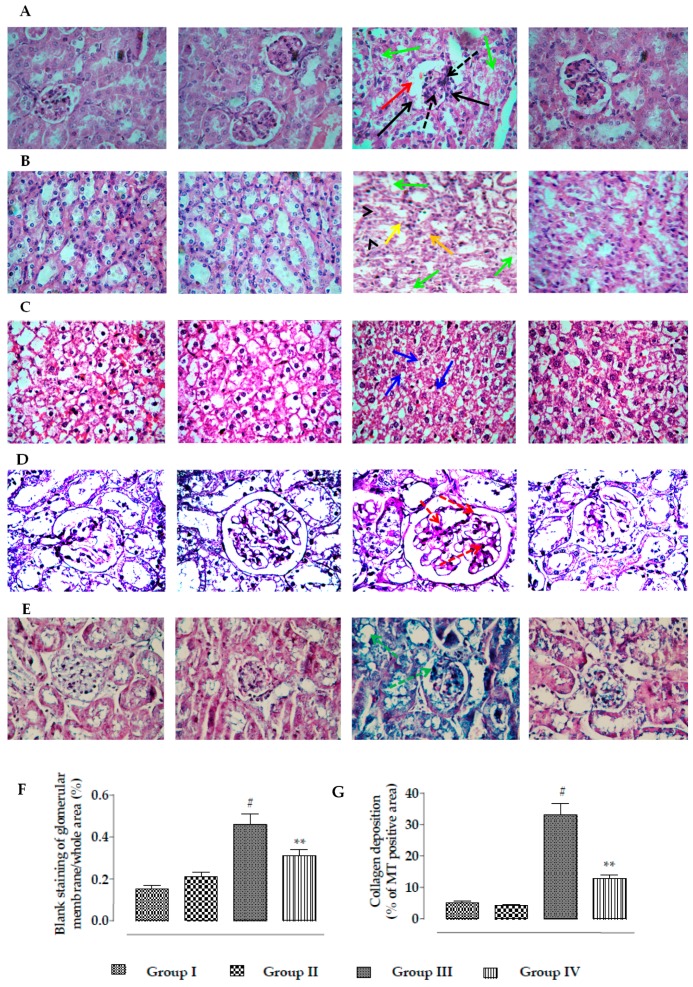
Histological assessments of kidneys of experimental mice in the absence (CdCl_2_) and presence of RA (CdCl_2_ + RA). (**A**) The H&E-stained sections of mice kidneys (×100), (**B**) H&E-stained medulla portion of mouse kidney, (**C**) H&E-stained cellular structure (×400) of mouse kidney, (**D**) PAS-stained sections of mice kidneys (×100), and (**E**) MT-stained sections of mice kidneys (×100). Red arrows indicate thickening of Bowman’s capsules; green arrows indicate cloudy appearance of tubules; arrow heads indicate degeneration of tubules; yellow arrows indicate vacuolation; black arrows indicate glomerular fibro-cellular crescent; black dotted arrows indicate mesangial hypercellularity; blue arrows revealed induction of apoptosis to the renal cells; red dotted arrows indicate glomerular mesangial matrix expansion; green dotted arrows indicate collagen deposition. (**F**) The widening of capsular space is shown as a percentage of the blank staining of glomerular membrane compared to the whole area of the photomicrograph (100×, arbitrarily selected areas containing one glomerulus). (**G**) Histo-quantification of collagen deposition in MT-stained kidney sections. Results are expressed as means ± SD (*n* = 30). ^#^ Values significantly (*p* < 0.01) differ from normal control. ** Values significantly (*p* < 0.01) differ from CdCl_2_ control.

Representative histological sections of kidneys were stained with H&E, PAS, and MT (Figure 12). The histological sections of the H&E-stained mouse kidney cortex of CdCl_2_-treated mice revealed thickening of Bowman’s capsules (red arrow), mesangial hypercellularity (black dotted arrow), glomerular fibro-cellular crescent (black arrows), and cellular damage with cloudy appearance of tubules (green arrows) when compared with normal mice (Figure 12A). H&E staining of the medulla portion of CdCl_2_-treated mouse kidneys showed a cloudy appearance (green arrows), degeneration (arrow heads), and vacuolation (yellow arrows) of the renal tubules (Figure 12B). H&E staining of mouse kidney revealed morphological changes, such as condensation and fragmentation of the nucleus, which revealed the induction of apoptosis (blue arrows) in renal cells (Figure 12C). On the other hand, RA (50 mg/kg) treatment significantly reversed CdCl_2_-mediated alteration of renal histological structures and restored renal histology to near-normal status. PAS-positive staining indicated glomerular mesangial matrix expansion (red dotted arrows) in the kidneys of CdCl_2_-treated mice (Figure 12D). On the other hand, RA (50 mg/kg) treatment significantly reversed CdCl_2_-mediated glomerular mesangial matrix expansion. MT staining revealed significant collagen deposition (green dotted arrows) in the kidneys of CdCl_2_-treated mice (Figure 12E). On the other hand, RA (50 mg/kg) treatment significantly attenuated CdCl_2_-mediated deposition of collagen. In morphometric analysis, widening of Bowman’s capsules was measured in H&E stained kidney sections (100×) taking the arbitrarily selected areas containing one glomerulus. The kidney sections of Cd-treated mice exhibited a significant (*p* < 0.01) widening of capsular space (Figure 12F). On the other hand, RA (50 mg/kg) treatment could significantly (*p* < 0.01) reciprocate the widening of Bowman’s capsules to near normal status (Figure 12F). Histo-quantification of MT-stained kidney sections showed that Cd caused significant (*p* < 0.01) enhancement in the collagen deposition within the renal tissue (Figure 12G). On the other hand, RA (50 mg/kg) treatment could significantly (*p* < 0.01) reduce the collagen deposition within the renal tissue (Figure 12G). In this study, RA (50 mg/kg) alone could not impart any change in the histological structure of kidneys, the glomerular matrix expansion, and the collagen deposition in kidneys.

### 2.3. Predictive Pharmacology In Silico

#### 2.3.1. Analyses of ADMET and Drug-Likeness Prediction 

The in silico analyses of drug-likeness and physicochemical properties, ADME profiles, water solubility (LogS scale), and pharmacokinetics profiles (gastrointestinal absorption) of RA are represented in Table 3. Lipinski’s rule of five was crucially measured to determine the drug-likeness profile of RA. Other important physico-chemical parameters, such as number of rotatable bonds = 7 (recommended value ≤10) and molar refractivity (MR) = 91.4 (recommended value 40–130) for RA, were also found to be well within the acceptable range. OSIRIS Property Explorer predicted the toxicity risks assessment profiles, which revealed that RA did not show any indication of tumorigenicity, mutagenicity, irritating effect, and reproductive toxicity in the in silico prediction. The above findings explained that RA has potential drug-likeness properties with numerous pharmacological implications.

#### 2.3.2. In Silico Binding Interaction Analysis through Molecular Docking Study

A molecular docking study of RA at the active site of the target proteins was analyzed with several parameters, such as Glide-based docking score, hydrogen-bond interaction, hydrophobic interaction, pi–pi (π–π) stacking interaction, π–cation, and salt-bridge formation; these were critically checked to envisage the binding affinities and anticipate the possible molecular alignment map (Table S1, Supplementary Materials). In silico molecular docking studies predicted the possible interactions of RA with Bad, Bcl-2, cyt c, Apaf1, caspase 9, caspase 3, caspase 8, IκBα, NF-κB, PKC-δ, TNFR2, TGF-β1, SMAD3, and E-cadherin. Selective receptors/proteins were retrieved from the publicly available Protein Data Bank (Figure S1, Supplementary Materials).

## 3. Discussion

Cd is one of the most hazardous natural and occupational pollutants and is considered to be a major threat to global health in the industrialized and agro-based countries around the world [6]. Continuous liberation of this non-biodegradable heavy metal from natural sources coupled with the increasing deliverance of bivalent Cd compounds through various anthropogenic activities resulted in an alarming increase of the Cd burden in the environment [16]. Numerous epidemiological reports revealed that Cd causes a number of health risks to humans and animals [6,12,16]. Considering the inherent association between Cd-mediated generation of oxidative free radicals and renal damage, the present study was commenced to assess the possible therapeutic potential of RA against Cd-induced nephrotoxicity. 

Earlier investigations exposed that Cd promotes ROS generation in the renal cells through a number of indirect mechanisms, such as disruption of electron flow in mitochondrial respiratory chain, releases of transition metals, and inactivation of endogenous redox defense molecules via binding to the sulfhydryl groups [6,16]. In this study, significant upregulation in the intracellular ROS production within Cd-exposed renal cells both in in vitro and in vivo systems was in accordance with earlier observations [6,11,12,17]. A critical level of ROS is crucial for maintaining normal cellular homeostasis [18]; however, the abnormal rise in the levels of various ROS in the Cd-exposed renal cells triggered oxidative damage to the cellular macromolecules, evidenced from the escalation in the extents of lipid peroxidation, protein carbonylation, DNA oxidation, and DNA fragmentation in the renal cells. On the other hand, RA treatment significantly reciprocated Cd-mediated increase in intracellular ROS levels, thereby reducing oxidative damage to the cellular biomolecules. These effects might be correlated to the direct hindrance of ROS production by RA [19,20] coupled with RA’s radical-scavenging effect [21].

Cellular antioxidant molecules serve as a first line of defense homeostasis to maintain a normal redox atmosphere via transferring electrons to the oxidative free radicals [18]. SOD scavenges superoxide radicals by converting them into molecular oxygen and hydrogen peroxide, which is subsequently reduced into water and oxygen; this reaction is catalyzed by CAT [1,22]. GST catalyzes the ROS-scavenging reaction of GSH [1], while GR promotes the reduction of GSSG into GSH [1]. GPx accelerates the consumption of peroxides via the reaction with GSH. G6PD is responsible for restoring NADPH concentration, which is necessary for GR activity [23]. Under normal physiological conditions, GR catalyzes the reduction of GSSG into GSH by NADPH and forms a normal redox cycle. In this study, CdCl_2_ caused significant depletion in the levels of antioxidant enzymes, which might be due to the strong affinity of Cd toward protein-bound sulfhydryl groups [9]. In this study, Cd-mediated increase in the level of NADPH oxidase resulted in the depletion of NADPH, which can also disrupt the normal redox cycle, resulting in the depletion of cellular GSH. On the other hand, RA treatment significantly reversed Cd-mediated depletion in the levels of the aforementioned antioxidant molecules. RA was reported to trigger the activation of nuclear factor E2-related factor 2 (Nrf2) signaling in redox-challenged human hepatic cells [24,25], which simultaneously activates phase II antioxidant enzymes [26]. Therefore, RA-mediated activation of endogenous antioxidant enzymes in the renal cells may be mediated through Nrf2 activation. On the other hand, RA-mediated activation of phase II antioxidant enzymes coupled with impairment of NADPH oxidase by RA could be attributed to the enhancement of GSH level in the renal cells. Therefore, it could be concluded that RA can protect the renal cells from Cd-mediated oxidative insults not only by free-radical quenching but also by boosting the cellular redox defense.

Oxidative free radicals were reported to activate apoptosis by modulating the expressions of different signal proteins of the apoptotic machinery [27]. Oxidative stress upregulates p53 and JNK, resulting in activation of Bad [28]. Bad, a pro-apoptotic member of the Bcl-2 family, synchronizes apoptosis via mitochondrial stress, involving its translocation from the cytosol to mitochondria [29]. Oxidative free radicals were reported to endorse depolarization of the mitochondrial membrane and open the channels for pro-apoptotic factors [28]. Anti-apoptotic Bcl-2 protein is mainly localized in the outer mitochondrial membrane, where it forms a heterodimer with Bad, thereby inhibiting the pro-apoptotic effect of Bad [28]. Additionally, p38 is a member of the MAPK family, which was revealed to inhibit Bcl-2 through direct interaction [30]. In this study, Cd significantly triggered p53/p38/JNK activation (Figure S2, Supplementary Materials) in the kidney cells and this could be correlated to the Cd-mediated augmentation in ROS production in the renal cells. Activation of p53/p38/JNK-MAPKs could further activate and impair pro-apoptotic and anti-apoptotic signal transduction, respectively. In this study, a significant downregulation of Bcl-2 and a significant upregulation of Bad expression were recorded in the Cd-exposed renal cells. On the other hand, RA significantly reciprocated Bad/Bcl-2 signaling in the renal cells, which could be correlated to the impairment of p53/p38/JNK activation via antioxidant effect of RA. In addition, oxidative stress can promote osmotic swelling of the mitochondrial matrix and enhance mitochondrial membrane permeability, which leads to the release of cyt c into the cytosol. In the cytosol, cyt c forms the apoptosome by complexing with Apaf-1 and procaspase 9 [28], which in turn results in auto-activation of caspase 9 and executes downstream effectors of the apoptotic cascade (caspase 3) [28]. In this study, Cd exposure significantly activated cytosolic release of cyt c, Apaf-1 expression, and cleavage of caspases in the renal cells. On the other hand, RA significantly reciprocated up- and downstream effectors of the apoptotic cascade, which may be due to the impairment of cyt c release via direct inhibition of osmotic swelling of the mitochondrial matrix, and RA also improved mitochondrial membrane integrity via the reduction of Cd-provoked oxidative stress through its antioxidant/radical-scavenging effect. It was revealed that oxidative free radicals can simultaneously execute death receptor-mediated apoptosis via activation of FAS and TNFR2 signaling [31,32]. Activation of FAS and TNFR2 endorses its downstream effector caspase 8 and triggers its cleavage, which in turn can cleave BH3-only protein Bid to form t-Bid [28]. Then, t-Bid translocates to the outer mitochondrial membrane, where it amplifies an intrinsic apoptotic event by triggering the mitochondrial translocation of pro-apoptotic members of the Bcl-2 family [33]. In this study, Cd exposure significantly activated death receptor-mediated apoptosis in the renal cells, evidenced from the over-expression of FAS, cleaved caspase 8, t-Bid, and TNFR2 (p75). On the other hand, RA significantly reciprocated death receptor-mediated apoptosis. 

NF-κB is a redox-sensitive transcription protein, which is also triggered by TNFR2 [34]. NF-κB signaling begins with the dissociation of NF-κB from its inhibitory IκBα via Ser phosphorylation followed by nuclear translocation of phospho-NF-κB [16]. Nuclear translocation of phospho-NF-κB was reported to trigger a pro-apoptotic event via promotion of Bad translocation into the mitochondria. In addition, NF-κB can also endorse FAS expression, thereby exerting a pro-apoptotic role [16]. Oxidative free radicals were reported to activate PKC isoforms, which can trigger NADPH oxidase and subsequently endorse ROS generation [35]. In this study, Cd treatment significantly upregulated NF-κB/PKC-δ signaling, evidenced from the significant over-expressions of phospho-IκBα (cytosol), phospho-NF-κB (nucleus), and PKC-δ (cytosol). On the other hand, RA significantly reciprocated NF-κB signaling, which may be correlated to the antioxidant/radical-scavenging effect of RA. NO is a critical vasodilator, which was found to be involved in the structural lesions to the tubular epithelial cells of the kidneys [17]. Cd can endorse cytotoxic effects to the renal region, mediated through nitric oxide synthesis via activation of iNOS [11]. In this study, Cd treatment significantly upregulated iNOS expression (Figure S2, Supplementary Materials), while, RA could significantly reciprocate iNOS expression to near-normal status.

TGF-β1/SMAD signaling plays an important role in the progress of renal fibrosis by triggering myofibroblast differentiation, extracellular matrix deposition, and renal epithelial–mesenchymal transition [11,36]. In addition, TGF-β1 can trigger MAPKs [37]. SMAD3 is the downstream target of TGF-β1, which controls the signal transduction mediated by its phosphorylation. TGF-β1-inhibitory SMAD, SMAD7, is regarded to be the key regulator of TGF-β1 signaling via a “negative feedback loop” [37]. Downregulated expression of Smad7 was reported to endorse TGF-β1-mediated renal fibrosis and inflammation [38,39]. Oxidative free radicals were reported to be involved in TGF-β1-induced renal myofibroblast differentiation and α-SMA expression [11]. E-cadherin is a component of adhesive connections between cells, which negatively regulates TGF-β1 activity, thereby inhibiting TGF-β1-mediated epithelial–mesenchymal transition [17]. TGF-β1 can trigger collagen gene expression and potentiate collagen deposition [40]. In this study, Cd treatment caused significant upregulation in TGF-β1, α-SMA, phospho-SMAD3, and collagen IV expressions, while significant downregulation in the SMAD7 and E-cadherin expressions was found in the Cd-treated renal cells. On the other hand, RA could significantly reciprocate Cd-mediated changes in TGF-β1, α-SMA, phospho-SMAD3, collagen IV, SMAD7, and E-cadherin expressions.

Abnormalities in the serum and urinary biochemical parameters give the primary impression of toxicological proceedings in the kidneys. A unique correlation between dyslipidemia and renal pathogenesis was reported in the patients with chronic renal diseases [41]. Dysregulation of several key enzymes and receptors involved in the lipid metabolism endorses renal pathogenesis [42]. In this study, significant enhancement in the level of cholesterol and triglycerides was observed in the sera of Cd-treated mice. LDH and CK are two membrane-bound enzymes widely distributed in various tissues [6]. Increased levels of LDH and CK give the impression of cell damage [16]. In this study, significant enhancement in the levels of LDH and CK in the sera of Cd-treated mice revealed loss of membrane integrity and cell damage. Elevated levels of urea, uric acid, and creatinine in the sera revealed reduction in glomerular filtration rate [43,44]. In this study, significant enhancement in the levels of urea, uric acid, and creatinine in the sera of Cd-treated mice revealed a decline in renal function. The reduced level of urinary creatinine and the high concentration of serum creatinine exposed the inhibition of creatinine clearance, which reveals an impairment of renal functions [29]. Release of urinary albumin is one of the prognostic indicators of renal pathogenesis, which occurs due to impairment of glomerular albumin filtration and/or albumin reabsorption in the renal tubules [45]. In this study, significant enhancement in the level of urinary albumin coupled with reduction of creatinine clearance in Cd-treated mice further revealed the nephrotoxic effect of Cd. On the other hand, RA treatment significantly reciprocated Cd-mediated abnormalities in serum and urinary parameters. The kidney is regarded to be the most critical target of Cd, where Cd is mostly bio-accumulated [6]. In this study, CdCl_2_ treatment caused significant enhancement of Cd burden in the kidneys. On the other hand, RA treatment significantly reduced renal Cd burden, which may be due to the promotion of Cd clearance by RA through its ability to chelate bivalent metals [13].

Cd treatment significantly enhanced NO level in the renal tissue, which is due to enhanced iNOS signaling. Increased NO level resulted in the activation and infiltration of macrophages and neutrophils, thereby participating in the process of renal pathology resulting in a loss of functional activity [11,46]. Activation of macrophages can induce inflammatory damage via release of pro-inflammatory mediators. In addition, NF-κB can serve as a pivotal mediator of inflammatory responses [47]. Activation of NF-κB executes its downstream pro-inflammatory mediators and promotes the releases of pro-inflammatory mediators from the macrophages [48]. In this study, significantly high levels of C-reactive proteins, TNF-α, IL-1β, and IL-6 were observed in the sera of Cd-treated mice, which revealed the establishment of inflammation. On the other hand, RA treatment significantly reduced Cd-mediated inflammatory stress, which would be correlated to the impairments of NO production and NF-κB activation.

The in silico docking-based molecular interaction maps between RA and several important signal proteins anticipated some specific key interactions and shed light on the orientation of the interaction mode which possibly occurs in a biological environment. Chemometric analysis predicted different types of molecular interactions at the active site of RA with Bad, Bcl-2, cyt c Apaf 1, caspase 9, caspase 3, caspase 8, IκBα, NF-κB, PKC- δ, TNFR2, TGF-β1, SMAD3, and E-cadherin. Hence, these interactions possibly trigger various signal transductions, which were observed in our in vitro and in vivo studies. In addition, in silico ADME and toxicity profile analyses predicted the drug-likeness characteristics of RA. The above findings anticipated that RA could be a potential drug candidate to exhibit multi-target therapeutic interventions in future.

## 4. Materials and Methods

### 4.1. Chemicals, Reagents, and Solvents

Cadmium chloride (CdCl_2_), fetal bovine serum (FBS), Bradford reagent, APO transferrin, hydrocortisone, antibiotic antimycotic solution, and bovine serum albumin (BSA) were obtained from Sigma-Aldrich Chemical Company, St. Louis, USA. Dulbecco’s modified Eagle’s/Ham’s F12 (DMEM-F12) and Hank’s balanced salt solution (HBSS) were procured from Thermo Fisher Scientific, USA. Antibodies for immunoblotting were obtained from Cell Signaling Technology, Beverley, USA, Denver, USA, and Bio-Rad, Hercules, USA. Furthermore, 5,5-dithiobis(2-nitrobenzoic acid), 1-chloro-2,4-dinitrobenzene, (NH_4_)_2_SO_4_, 2,4-dinitrophenylhydrazine, 5-thio-2-nitrobenzoic acid, CCl_3_COOH, ethylene diaminetetraacetic acid, Na_4_P_2_O_7_, NaN_3_, KH_2_PO_4_, *N*-ethylmaleimide, nitro blue tetrazolium, phenazinemethosufhate, reduced glutathione, reduced nicotinamide adenine dinucleotide (NADH), and thiobarbituric acid were purchased from Sisco Research Laboratory, Mumbai, India. HPLC-grade solvents were brought from Merck, Mumbai, India. Kits for measurement of different biochemical parameters were purchased from Span diagnostic Ltd., India.

### 4.2. Animals

Healthy Swiss albino mice (♂, 4 ± 1 week old, 25 ± 5 g) were procured from Chakraborty Enterprise, Kolkata, India. Animals were kept in standard polypropylene cages in the animal house of the Department of Pharmaceutical Technology, Jadavpur University, India, and they were maintained with a temperature of ~24 °C, a humidity of ~45%, and a 12-h light/dark cycle [49]. The mice were fed a standard diet and water ad libitum. The experiment was approved (Ref no.: AEC/PHARM/1701/01/2017) by the Institutional Animal Ethical Committee (Registration no.: 0367/01/C/CPCSEA, UGC, India). The study was also approved by Committee for the Purpose of Control and Supervision on Experiments on Animals. The principles of laboratory animal care were followed throughout the animal experiment [50].

### 4.3. In Vitro Bioassays

#### 4.3.1. Isolation of Mouse Proximal Tubular Epithelial Cells

Mouse proximal tubular epithelial cells were isolated following the standard protocol [51]. Briefly, mice were sacrificed by cervical dislocation under CO_2_ euthanasia. Kidneys were excised and immediately transferred into ice-cold (0 °C) Hank’s balanced salt solution (HBSS) fortified with 1% antibiotics. The kidney was longitudinally cut into two halves and the medulla was discarded. The cortical tissue was crushed and transferred to 10 mL of HBSS containing collagenase. Tubules were incubated at 37 °C at 70 rpm for 30 min with a gentle mixing after 15 min. Following digestion, the density sedimentation was done to inactivate enzymes using heat-inactivated horse serum. After this step, tubules were washed once with HBSS and centrifuged at 500× *g* for 5 min. The sediment was re-suspended in kidney culture media as described by Breggia and Himmelfarb [51]. Then, 200 µL of tubular cell suspension containing 1 × 10^6^ cells was transferred into each well of a 24-well 1% gelatin-coated cell culture plate and incubated at 37 °C and 5% CO_2_. Culture media was replaced initially after 24 h and then every 72 h using kidney culture media without recombinant human epidermal growth factor. After one week, cell cultures were organized as a confluent monolayer for subsequent assays.

#### 4.3.2. Determination of Cytotoxic Effect of CdCl_2_ on Mouse Proximal Tubular Epithelial Cells

The concentration-dependent cytotoxic effect of CdCl_2_ on mouse proximal tubular epithelial cells was determined employing MTT (3-(4,5-dimethylthiazolyl-2)-2, 5-diphenyltetrazolium bromide) assay [52]. Seven-day-old adherent tubular epithelial cells were treated with CdCl_2_ (0.05–1000 µM) for 24 h and the cell viability was measured using the MTT assay. Briefly, 250 µL of MTT solution (300 mg/mL) was added to the culture medium (200 µL/well) 1 h before the end of the 24-h treatment and incubated for 30 min. After incubation, supernatants were discarded, and 200 µL of DMSO was added and absorbance was measured at λ_excitation_ 570 nm/λ_emission_ 630 nm. The differences between the absorbance and a percentage of the corresponding controls were used to express cell viability. The experiment was repeated thrice. The normalized cell viability data were represented. CdCl_2_ exhibited an IC_50_ value of 42.8 µM (~40 µM) in murine proximal tubular epithelial cells. An earlier report revealed that CdCl_2_ can impart apoptosis at the dose of 20 µM as early as 18 h to renal proximal tubule epithelial cells [53]. Considering this, we chose a high dose of CdCl_2_ of 40 μM (~IC_50_) for subsequent in vitro assays to evaluate anti-apoptotic mechanism of RA.

#### 4.3.3. Determination of Cytoprotective Role of RA Against CdCl_2_

To determine the in vitro cytoprotective effect of RA, seven-day-old adherent tubular epithelial cells in a 24-well cell culture plate were treated with RA (5–60 µM) 1 h prior to CdCl_2_ (40 µM) treatment and then incubated for 24 h. The cell viabilities were measured employing the MTT assay at different intervals up to 24 h [52]. The normalized cell viability data were represented.

#### 4.3.4. Hoechst Nuclear Staining

For Hoechst staining, 50 µL of a tubular cell suspension containing 2 × 10^3^ cells was transferred into each well of a 96-well cell culture plate and maintained as mentioned earlier. Seven-day-old adherent tubular epithelial cells were treated with CdCl_2_ (40 µM) alone, RA (40 µM) alone, and RA (40 µM) 1 h prior to CdCl_2_ (40 µM) treatment, and then incubated for 24 h at 37 °C and 5% CO_2_. One untreated set served as a normal control. After 24 h, cells were fixed with paraformaldehyde (4%) in phosphate-buffered saline (PBS) of pH 7.4 for 20 min [54]. Then, the tubular sets were strained with Hoechst 33258 (5 μg/mL in PBS) for 20 min followed by washing with PBS [54]. Fluorescent nuclei and nuclear pattern were noted.

#### 4.3.5. Flow Cytometric Analysis

The flow cytometric analysis was executed to reveal the type of cell death. Briefly, seven-day-old mouse proximal tubular epithelial cells were exposed to CdCl_2_ (40 µM) alone, RA (40 µM) alone, and RA (40 µM) 1 h prior to CdCl_2_ (40 µM) treatment, and then incubated for 24 h at 37 °C and 5% CO_2_. One set of untreated cells was kept as a normal control. After 24 h, different sets of cells were treated with propidium iodide (PI) and FITC-labeled annexin V for 30 min [55]. The excess of PI and annexin V was washed out, and the cells were fixed for analyzing in a flow cytometer using FACS Calibur (Becton-Dickinson, Mountain View, USA) equipped with a 488-nm argon laser light; a 515-nm band pass filter was used for FITC fluorescence, and a 623-nm band pass filter was used for PI fluorescence. The scatter plots of PI fluorescence (*y*-axis) vs. FITC fluorescence (*x*-axis) were prepared for different sets of treatments.

#### 4.3.6. Oxidative Stress Analyses In Vitro

Seven-day-old mouse proximal tubular epithelial cells were exposed to CdCl_2_ (40 µM) alone, RA (40 µM) alone, and RA (40 µM) 1 h prior to CdCl_2_ (40 µM) treatment, and then incubated for 24 h at 37 °C and 5% CO_2_. One set of untreated cells was kept as a normal control. After 24 h of treatment, the intra-cellular ROS production was assessed under fluorescence microscope (Olympus-1X70, Japan, software-Metamorph) using 2^’^,7^’^-dichlorofluorescein diacetate (DCF-DA), which quantitatively reacts with ROS and oxidizes to a fluorescence dye, 2^’^,7^’^-dichlorofluorescein (DCF) [56]. The cellular level of nitric oxide (NO) was assayed by a colorimetric assay using a commercially available assay kit and following the manufacturer’s protocol (Cayman Chemical Company, Ann Arbor, MI, USA). H_2_O_2_ content was measured by the methods described by Fraga and co-workers [57]. NADPH oxidase activity was analyzed by the method described by Herrera and co-workers [58]. The extent of lipid peroxidation was assayed by quantifying the level of cellular thiobarbituric acid-reactive substances (TBARS) as per an established protocol [57]. The protein carbonylation was determined in accordance with the standard protocol [59]. The levels of co-enzymes Q9 and Q10 within the cell lysate were separated and quantified by RP-HPLC (Dionex, Germany) analyses as described earlier by Zhang and co-workers [60]. The levels of the endogenous antioxidant enzymes, such as superoxide dismutase (SOD), catalase (CAT), glucose-6-phosphate dehydrogenase (G6PD), glutathione peroxidase (GPx), glutathione-*S*-transferase (GST), and glutathione reductase (GR) were measured following methods described elsewhere [61]. Reduced (GSH) and oxidized (GSSG) glutathione contents in the renal cells were determined following the method developed by Hissin and Hilf [62].

#### 4.3.7. Immunoblotting of Signal Proteins in Vitro

Seven-day-old mouse proximal tubular epithelial cells were exposed to CdCl_2_ (40 µM) alone, RA (40 µM) alone, and RA (40 µM) 1 h prior to CdCl_2_ (40 µM) treatment, and then incubated for 24 h at 37 °C and 5% CO_2_. One set of untreated cells was kept as a normal control. After 24 h of treatment, the nuclear, cytosolic, and mitochondrial protein fractions of mouse proximal tubular epithelial cells were separated following a standard sequential fractionation procedure as described by Baghirova and co-workers [63]. The sample proteins (20 µg) were resolved in 10% SDS-PAGE gel electrophoresis and transferred into a nitrocellulose membrane [55]. The membrane was washed with Tris-buffered saline containing 0.1% Tween-20 (TBST) and blocked for 1 h using a blocking buffer containing 5% non-fat dry milk. After blocking, the membrane was incubated with primary antibody at 4 °C overnight. The membrane was then washed with TBST and was treated with a suitable HRP-conjugated secondary antibody at room temperature for 1 h. The blot was developed by ECL substrate (Millipore, MA, USA) for the detection of protein expressions in a ChemiDoc touch imaging system (Bio-Rad, USA). The densitometric analysis was performed using Image Lab software (Bio-Rad, USA). Normalization of expression was done with respect to β-actin. The membranes were then subjected to mild stripping to detect the expressions of other proteins in the same membrane [49]. The expressions of Bcl-2, Bax, Bad, cytochrome c (cyt c), Apaf-1, cleaved caspase 9, cleaved caspase 3, t-Bid, FAS, cleaved caspase 8, phospho-IκBα (Ser 32), total IκBα, phospho-NF-κB (p65) (Ser 536), PKC-δ, TNFR2, total JNK, phospho-JNK (Tyr 183/Tyr 185), total p38, phospho-p38 (Tyr 180/Tyr 182), p53, iNOS, TGF-β1, α-SMA, phospho-SMAD3 (Ser 423/Ser 425), total SMAD3, SMAD7, E-cadherin, and collagen-IV were studied.

### 4.4. In Vivo Bioassay

#### 4.4.1. Experimental Set-Up

After two weeks of acclimatization with the experimental atmosphere of temperature (~24 °C), humidity (~45 %), and 12-h light/dark cycle, 24 Swiss albino mice were randomly divided into four groups (*n* = 6) and the animals received the following treatments:

Group I: Normal control (animals received only 1% Tween-80 in distilled water as a vehicle);

Group II: Animals were treated with RA (50 mg/kg body weight, p.o.) for 14 days; a fresh dose of RA was prepared by dispersing it into double-distilled water containing 1% Tween-80 prior to daily dosing;

Group III: Toxic control (animals received CdCl_2_ through double-distilled water containing 1% Tween-80 at a dose of 4 mg/kg body weight, p.o. for 14 days, once daily);

Group IV: Animals were treated with RA (50 mg/kg body weight, p.o.) 1 h prior to CdCl_2_ (4 mg/kg body weight, p.o., once daily) treatment for the next 14 days. In this group, we used RA dispersed in double-distilled water containing 1% Tween-80 and CdCl_2_ dissolved in only double-distilled water to maintain the quantity of Tween-80 intake at the level of the individual group.

**Figure 13:**
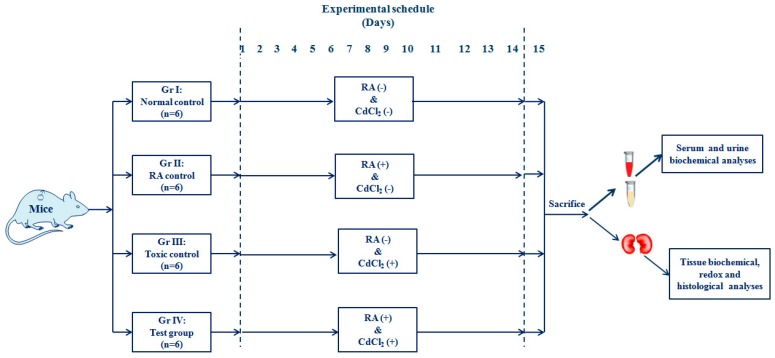
A schematic impression of in vivo experimental protocol.

The dose of CdCl_2_ was chosen on the basis of the LD_50_ value of CdCl_2_ (~78 mg/kg, p.o., probits value), and the previous experiments being conducted by the group [1,6]. The dose of RA was chosen on the basis of a preliminary study with a limited number of mice (*n* = 3). The in vivo experimental scheme is depicted in Figure 13.

After 14 days of treatment, the mice were fasted overnight and sacrificed on day 15 by cervical dislocation under CO_2_ euthanasia. Before sacrificing, the blood samples were collected from the retro-orbital venous plexus after applying tetracaine (0.5%, one drop) ophthalmic anesthetic drop to the eyes. To measure urinary parameters, urine samples from different groups of animals were collected from the bladder and immediately stored at −80 °C. The kidneys were excised and were instantaneously cleaned with PBS. The excised kidneys were differently processed for different analyses. The excised kidneys were homogenized immediately in Tris-HCl (0.01 M)–EDTA (0.001 M) buffers of pH 7.4 and centrifuged at 12,000× *g* for 30 min at 4 °C to obtain supernatants for subsequent biochemical analyses [64]. For immunoblot analyses, the excised kidneys were subjected to a sequential fractionation procedure to obtain specific cellular fractions [63]. For histological analysis, the kidney parts were immediately fixed in 10% buffered formalin [64].

#### 4.4.2. Estimation of Serum and Urine Biochemical Parameters

The levels of total cholesterol, HDL cholesterol, triglycerides, creatine kinase (CK), lactate dehydrogenase (LDH), urea, uric acid, creatinine, and C-reactive protein in the sera were estimated using a commercially available assay kit and following the manufacturer’s protocol (Span Diagnostic Limited, India). The creatinine, albumin, and nitrate/nitrite levels in the urine were measured using colorimetric assay kits (Span Diagnostic Limited, India). Serum TNF-α, IL-1β, and IL 6 contents were analyzed by ELISA kits (Fisher Thermo Scientific Co., USA).

#### 4.4.3. Estimation of Cd Contents in Renal Tissue

Cd content in renal tissue was estimated following the protocol of Pari and co-workers [63]. Briefly, a portion of renal tissue was digested with a mixture of de-ionized water, nitric acid, and hydrogen peroxide to near dryness. The dried mass was further dissolved in 1% nitric acid, and Cd in the solution content was estimated by an atomic absorption spectrophotometer (Perkin Elmer Model no. 3100, US) using an appropriate cathode lamp.

#### 4.4.4. Oxidative Stress Analyses In Vivo

The levels of ROS, NO, H_2_O_2_, NADPH oxidase, co-enzyme Q9, co-enzyme Q10, SOD, CAT, G6PD, GR, GST, GPx, GSH, and GSSG in the kidneys of the mice receiving different treatments were measured following the established protocol mentioned earlier. The extent of DNA fragmentation in the renal cells was evaluated by the diphenylamine reaction, while DNA oxidation in the renal cells was measured by RP-HPLC (Dionex, Germany) analysis [49].

#### 4.4.5. Immunoblotting of Signal Proteins in Vivo

The expressions of Bcl-2, Bax, Bad, cytochrome C, Apaf-1, cleaved caspase 9, cleaved caspase 3, t-Bid, FAS, cleaved caspase 8, phospho-IκBα (Ser 32), total IκBα, phospho-NF-κB (p65) (Ser 536), PKC-δ, TNFR2, total JNK, phospho-JNK (Tyr 183/Tyr 185), total p38, phospho-p38 (Tyr 180/Tyr 182), p53, iNOS, TGF-β1, α-SMA, phospho-SMAD3 (Ser 423/Ser 425), total SMAD3, SMAD7, E-cadherin, and collagen-IV in specific cellular components were studied following the protocols mentioned earlier.

#### 4.4.6. Histological Analyses

Formalin-fixed kidneys of experimental mice were subjected to paraffin mounting followed by sectioning. Sections (~5 µm) were subjected to hematoxylin and eosin (H&E), periodic acid–Schiff (PAS), and Masson’s trichrome (MT) staining as per the established protocol [6,64,65]. Histo-quantification was performed using NIH IMAGE (Image-J, 1.37v) software.

### 4.5. Statistical Analysis

The data were represented as means ± SD. The data were statistically analyzed by one-way ANOVA followed by Dunnett’s *t*-test employing GraphPad InStat (version 3.05), Graph-Pad software, USA. The *p*-values less than 0.05 were considered significant.

### 4.6. In Silico Analyses

#### 4.6.1. In Silico ADMET and Drug-Likeness Prediction

Considering the RA as a good drug candidate, several important parameters, such as physico-chemical and drug-likeness properties, nature of lipophilicity, solubility, and other pharmacokinetics profiles, were computationally predicted. SwissADME [66], a web server-based prediction tool, developed and maintained by the SIB (Swiss Institute of Bioinformatics), was used for this purpose to predict and collect the information on absorption, distribution, metabolism, and excretion (ADME) and pharmacokinetic properties. Another standalone open source tool, OSIRIS Property Explorer [67], was used for theoretically predicting toxicity risk assessment for RA, which graded the predicted toxicity risk assessment in provisions of high, medium, and low toxic risk factors.

#### 4.6.2. In Silico Molecular Docking Analyses

Macromolecular structural data of selective receptors/proteins were retrieved from the publicly available Protein Data Bank (PDB), archived at www.rcsb.org [68,69]. High-resolution protein crystal structures were downloaded and were preprocessed with the help of the “Protein Preparation Wizard” of Schrödinger suite [70] for appropriate bond order assignment, hydrogen atom addition, repairing of missing loops, and deletion of water in the crystal structure. During the refinement process, different possible states of histidine amino-acid residues were assigned to all crystal structures. Finally, in order to obtain the minimum energy structural conformation of each protein, the system was subjected to minimization to an RMSD of 0.30 Å based on force field OPLS2005 applied. The “LigPrep” [70] module embedded in Schrödinger suite was used for generating low-energy conformations of the ligand, i.e., RA. Based on the applied force field, “OPLS_2005”, new conformations were acquired and possible ionization states for RA were generated at target pH 7.0 ± 2.0. Employing default settings in “LigPrep”, a pool of 32 stereoisomers was attained by retaining specified chiralities allowed for the ligand RA. Using the “Receptor Grid Generation” panel of Schrödinger suite [70], grid space was defined with the information of the coordinate of the bound ligand in the case of a PDB macromolecular complex attached with any bound co-crystallized ligand. Where no information of active sites of the protein was available, the SiteMap module [70] of Schrodinger suite was used to predict and identify the top-ranked potential receptor binding sites in the macromolecular structures. Following the receptor grid generation step for attaining the molecular docking simulation technique, the Glide module-based extra-precision docking (XP docking) method was employed, keeping the default settings. Only the customized selection was made to explore 10 docked poses per ligand.

## 5. Conclusions

**Figure 14:**
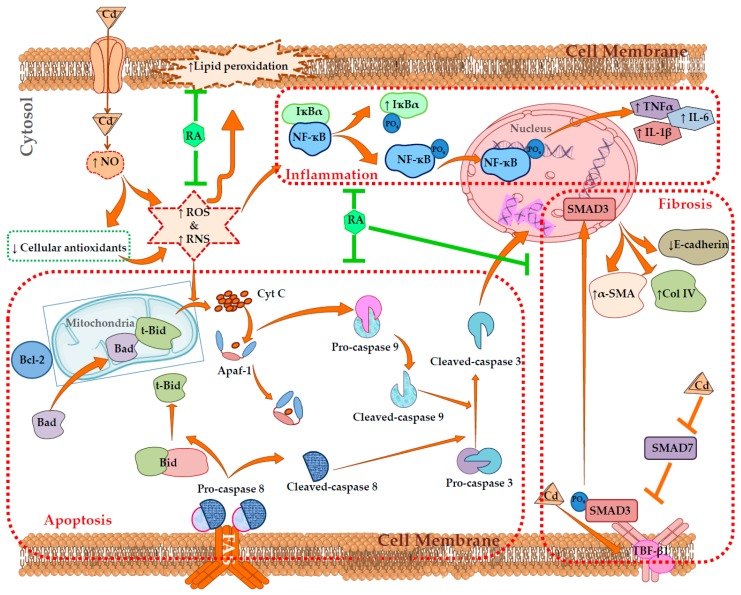
Schematic overview of probable protective mechanism of RA against Cd-mediated renal injury. The brown arrows (**→**) indicate downstream cellular events. The brown lines (T) indicate the activities restricted. The green lines (T) indicate the pathological event restricted by RA.

The present study implicated that CdCl_2_-mediated enhancement in the production of oxidative free radicals potentially contributes in the pathophysiology of mouse kidneys. Oxidative insult was found to endorse apoptotic and inflammatory signal transduction by triggering NF-κB/TNFR2/MAPK/PKC-δ activation in the renal cells. In addition, CdCl_2_ can induce renal fibrosis by endorsing TGF-β1/SMAD3/α-SMA/collagen signaling. On the other hand, RA treatment significantly attenuated Cd-mediated nephrotoxicity by scavenging oxidative free radicals, promoting cellular redox defense, accelerating Cd clearance, and reciprocating pathological signal transduction, evidenced from in vitro and in vivo observations (Figure 14). In silico molecular docking studies anticipated the possible interactions between RA and different signal proteins. In silico ADME and toxicity profile analyses predicted drug-likeness characteristics of RA. Therefore, RA can be a potential agent to treat Cd-mediated nephrotoxicity in future.

## Figures and Tables

**Table 1:** The effects on serum biochemical parameters in the absence (CdCl_2_) and presence of RA (CdCl_2_ +RA) in mice.

Parameters	Group I	Group II	Group III	Group IV
Total cholesterol (mg/dL)	82.12 ± 7.54	81.33 ± 8.92	138.76 ± 12.50 ^#^	118.22 ± 10.67 **
HDL cholesterol (mg/dL)	31.58 ± 2.87	31.12 ± 3.24	23.16 ± 2.33 ^#^	28.22 ± 2.41 *
Triglycerides (mg/dL)	91.15 ± 8.67	87.58 ± 8.52	118.21 ± 10.45 ^#^	104.11 ± 9.89 *
LDH (U/L)	161.48 ± 14.23	155.23 ± 15.92	238.21 ± 22.78 ^#^	184.55 ± 17.24 **
CK (IU/ mg of protein)	27.28 ± 2.44	26.02 ± 2.72	36.42 ± 3.38 ^#^	31.57 ± 2.98 *
Urea (mg/dL)	22.13 ± 1.78	23.02 ± 2.25	34.42 ± 3.08 ^#^	28.72 ± 2.77 **
Uric acid (mg/dL)	2.23 ± 0.25	2.03 ± 0.19	3.87 ± 0.29 ^#^	3.12 ± 0.31 **
Creatinine (mg/dL)	0.47 ± 0.05	0.45 ± 0.04	0.71 ± 0.08 ^#^	0.62 ± 0.06 *

Data are represented as means ± SD (*n* = 6). ^#^ Values significantly (*p* < 0.01) differ from normal control. * Values significantly (*p* < 0.05) differ from toxic control. ** Values significantly (*p* < 0.01) differ from toxic control.

**Table 2:** The effects on kidney mass, Cd accumulation, and renal function related to urine parameters.

Parameters	Group I	Group II	Group III	Group IV
Kidney mass (mg)	47.24 ± 4.54	45.67 ± 3.92	55.67 ± 6.12 ^$^	48.22 ± 5.01 *
Kidney mass/body mass (× 10^3^)	15.28 ± 1.48	13.94 ± 1.24	20.33 ± 2.17 ^#^	17.89 ± 1.31 *
Cd burden in kidney (ppm of wet tissue)	0.02 ± 0.001	0.007 ± 0.0008	20.33 ± 2.37 ^#^	17.89 ± 1.31 *
Urinary creatinine (mg/dL)	65.52 ± 6.33	64.33 ± 7.12	31.50 ± 3.21^#^	41.57 ± 3.98 *
Urinary albumin (mg/dL)	2.98 ± 0.24	2.78 ± 0.19	6.74 ± 0.75 ^#^	5.98 ± 0.49 *
Urinary nitrate/nitrite (nmol/g creatinine)	0.85 ± 0.09	0.82 ± 0.08	1.45 ± 0.16 ^#^	1.17 ± 0.12 **

Data are represented as means ± SD (*n* = 6). ^$^ Values significantly (*p* < 0.05) differ from normal control. ^#^ Values significantly (*p* < 0.01) differ from normal control. * Values significantly (*p* < 0.05) differ from toxic control. ** Values significantly (*p* < 0.01) differ from toxic control.

**Table 3:** In silico prediction of drug-likeness and ADMET profiles of RA.

Properties	Profile	Values
Properties under Lipinski’s rule of five	Molecular weight	360.31
	Hydrogen bond acceptor	8
	Hydrogen bond donor	5
	Octanol water coefficient (LogP)	2.36
Other physico-chemical properties	Number of rotatable bonds	7
	Molecular refractivity	91.4
	Topological polar surface area	144.52
Water solubility	Log *S*	Soluble
Pharmacokinetics profiles	GI absorption	Low
	log *K*p (cm/s)	−6.82
	CYP1A2 inhibitor	No
	CYP2C19 inhibitor	No
	CYP2C9 inhibitor	No
	CYP2D6 inhibitor	No
	CYP3A4 inhibitor	No
Drug-likeness profiles	Bioavailability score	0.55
Toxicity risk assessment	Mutagenicity risk	Low
	Irritating effect	Low
	Reproductive toxicity effect	Low
	Tumorigenicity risk	Low

## Supplementary Materials

Supplementary materials can be found at https://www.mdpi.com/1422-0067/20/8/2027/s1: Table S1. Dock score, emodel value, and interacting residues in molecular docking analysis of different bioactive proteins with RA; Figure S1. Docking interactions of RA with Bad (A), Bcl-2 (B), cyt c (C), Apaf1 (D), caspase 9 (E), caspase 3 (F), caspase 8 (G), IκBα (H), NF-κB (I), PKC-δ (J), TNFR2 (K), TGF-β1, (L), SMAD3 (M), and E-cadherin (N). Selective receptors/proteins were retrieved from the publicly available Protein Data Bank (PDB); Figure S2. The effects on MAPKs, and iNOS expressions in the absence (CdCl_2_) and presence of RA (CdCl_2_ + RA) in vitro (A) and in vivo (B). The relative band intensities were assessed, and the intensity of the normal control band was assigned as 1. β-Actin served as the loading control. Data are represented as means ± SD, *n* = 3 (in vitro) or *n* = 6 (in vivo). ^#^ Values significantly (*p* < 0.01) differ from normal control. * Values significantly (*p* < 0.05) differ from CdCl_2_ control. ** Values significantly (*p* < 0.01) differ from CdCl_2_ control.
