# Supplementary material for: Rosmarinic Acid Attenuates Cadmium-Induced Nephrotoxicity via Inhibition of Oxidative Stress, Apoptosis, Inflammation and Fibrosis
Source: Int J Mol Sci. 2019 Apr 24;20(8):2027. doi: 10.3390/ijms20082027 (PMC6514581; doi:10.3390/ijms20082027)
Supplement: Supplementary file 1 [file ijms-20-02027-s001.pdf]

Molecular docking

Molecular docking study of RA at the active site of the target proteins was analyzed with several parameters, such as Glide based docking score, hydrogen bond interaction, hydrophobic interaction, pi-pi (π-π) stacking interaction, π-cation, and salt bridge formation were critically checked to envisage the binding affinities and anticipating possible molecular alignment map (Supplementary table 1). The possible interactions of RA with Bad, Bcl-2, cyt c, Apaf1, caspase 9, caspase 3, caspase 8, IκBα, NF-κB, PKC-δ, TNFR2, TGF-β1, , SMAD3, and E-cadherin. Selective receptors/proteins were retrieved from the publicly available Protein Data Bank (Supplementary Figure 1). Molecular docking interactions between Bad and RA revealed involvement of Arg310, Arg313, and Asp317 amino acid residues in H-bond interactions. Apart from H-bond interactions, an oxygen atom participated in the salt bridge interaction with the positively charged Arg310 residue. Glide XP-based docking method revealed two numbers of H-bonds interactions between Bcl-2 protein and RA along with one π-π stacking interaction with Phe101 and one π-cation interaction with Arg143 residue. The cyt c and RA docking interaction revealed that Ile11, Lys27, and Tyr97 residues are involved in H-bond interaction along with a salt bridge formation with hydrophilic residue Lys27. Eight numbers of H-bond interactions with four consecutive amino acid residues Gly159, Lys160, Ser161, Val162, and two aspartate residues (Asp244, Asp392) have been observed in the docked conformations of Apaf1 and RA. The binding interactions between the Apaf1 and ligand RA also possess to form π-π stacking interaction with basic amino acid residue His438. The significance of π–π interaction effect might have contributing to overall binding energies for stabilizing the Apaf1 and RA molecular complex which was turned out with docking score of -9.91 Kcal/mol. Caspase 9 and RA docking complex suggested that Ser236, Gln285, and Arg355 residues were participated to form H-bond formation with RA. Moreover, two other non-covalent types of interactions were also found to form with two consecutive amino acid residues Trp354 (involved in π–π stacking) and Arg355 (involved in salt bridge formation). In the caspase 3-RA docking complex, two amino acid residues, Asn80 and His277 were found to be important in the H-bond interaction with -OH groups of RA. In addition, π-cation interaction was formed between the Lys82 residue of caspase 3 and catechol ring of RA. The molecular docking interaction analysis between caspase 8 and RA revealed H-bond interactions with Tyr334, Glu396, and Thr467 amino acid residues. Caspase 8 and RA docking interaction also suggested to form π-π stacking interaction with hydrophobic residue Phe399. IκBα and RA docking complex was formed via total four numbers of molecular interactions, such as three numbers of H-bonding with amino acid residues of Gln112, Thr121, and Glu153 and one salt bridge formation with IKBα residue of Arg143. The NF-κB and RA docking interaction revealed that Arg57, Arg59, His67, Asp274, and Lys275 residues are involved in H-bond. In addition, hydrophobic amino acid residue Phe56 formed π-π stacking interaction and Lys244 residue participated in salt bridge formation with RA. With PKC-δ protein, only a single amino acid residue was found to form two H-bond interactions with RA to obtain a moderate dock score value of −6.06 Kcal/mol. Although, a series of prominent hydrophobic amino acids (Pro26, Phe27, Cys28, Ala29, Tyr52, and Pro53 etc.) have been found in surrounding radius for showing hydrophobic interactions between RA and PKC-δ. The binding interactions of with TNFR2 showed the involvements of three amino acid residues (Ser107, Lys108 and Glu133) in H-bond formation with docking score -4.02 Kcal/mol. In silico docking analysis of TGF-β1 and RA revealed bit lower docking score of -3.91 Kcal/mol, producing three H-bond interactions with two polar amino acid residues (Asn42, Asn103), where Asn42 has participated to form double H-bond interactions with OH group in RA. The RA showed moderate binding affinity potential towards active site of SMAD3 protein, as observed from its docking score value of -6.20

Kcal/mol. The SMAD3-RA docking complex revealed that amino acid residues are at close proximity i.e. Thr366, Arg367 and a polar residue Gln315 of SMAD3 protein have found to form three H-bond interactions along with a salt bridge formation with common residue Arg367. The transmembrane glycoprotein, E-cadherin, produced total five numbers of H-bond interactions with the docking score of -5.44 Kcal/mol. Four negatively charged aspartate residues, such as Asp100, Asp136, Asp137, and Asp138 and a positively charged Arg68 residue have been found to participate in H-bond interaction. Here, the interesting point to be noticed in the molecular interaction map that all aspartate amino acid residues interacted with OH group of RA irrespective of their close proximity. One π-cation interaction is also found between cationic side-chain residue of Arg68 and E-cadherin protein, which clearly demonstrated the importance of this non-covalent binding interaction relevant for exhibiting necessary biological functions during ligand-protein interaction.

Supplementary Table 1. Dock score, emodel value and interacting residues in molecular docking analysis of different bioactive proteins with RA.

Sl. No.	Proteins	Glide docking score (Kcal/mol)	Glide emodel values	Interacting residues in H-bond interaction	Other interactions [pi-pi (π- π)/ pi-cation(π-cation)/salt bridge]
1	Bad	-4.50	-39.37	Arg310, Arg313, Asp317	Arg310 (salt bridge)
2	Bcl-2	-5.62	-44.44	Asn140, Gly142	Phe101 (π - π) / Arg143 (π -cation)
3	Cyt C	-5.81	-39.28	Ile11, Lys27, Tyr97	Lys27 (salt bridge)
4	Apaf1	-9.91	-89.33	Gly159, Lys160, Ser161, Val162, Asp244, Asp392	His438 (π - π)
5	Caspase 9	-6.20	-54.26	Ser236, Gln285, Arg355	Trp354 (π - π) / Arg355 (salt bridge)
6	Caspase 3	-4.81	-45.91	Asn80, His277	Lys82 (π -cation)
7	Caspase 8	-5.31	-45.49	Tyr334, Glu396, Thr467	Phe399 (π - π)
8	Iκ-Bα	-2.73	-26.73	Gln112, Thr121, Glu153	Arg143(salt bridge)
9	NF-κB	-6.01	-47.24	Arg57, Arg59, His67, Asp274, Lys275	Lys244 (salt bridge) / Phe56 (π - π)
10	PKC-δ	-6.06	-35.26	Ala23	-
11	TNFR2	-4.02	-41.03	Ser107, Lys108, Glu133	-
12	TGF-β1	-3.91	-40.15	Asn42, Asn103	-
13	SMAD3	-6.2	-40.04	Thr366, Arg367, Gln315	Arg367(salt bridge)
14	E-cadherin	-5.44	-42.44	Arg68, Asp100, Asp136, Asp137, Asp138	Arg68 (π-cation)

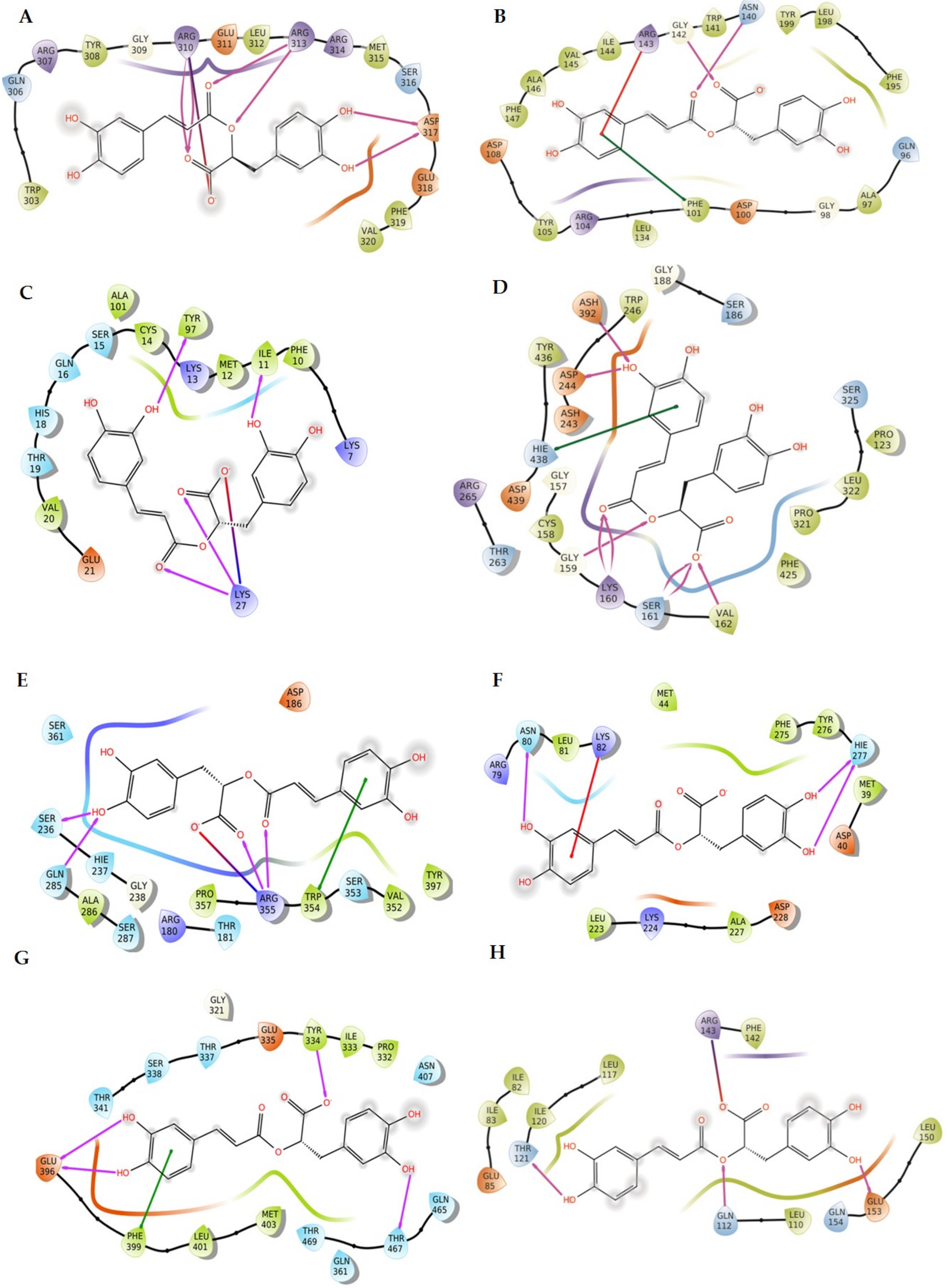
A
ARG 307
TYR 308
GLY 309
ARG 310
GLU 311
LEU 312
ARG 313
ARG 314
MET 315
SER 316
GLN 306
ASP 317
GLU 318
PHE 319
VAL 320
TRP 303
B
ASN 140
TYR 199
LEU 198
GLY 142
TRP 141
ARG 143
ILE 144
VAL 145
ALA 146
PHE 147
PHE 195
ASP 108
GLN 96
ALA 97
GLY 98
ASP 100
PHE 101
LEU 134
ARG 104
TYR 105
C
ALA 101
TYR 97
SER 15
CYS 14
LYS 13
MET 12
ILE 11
PHE 10
GLN 16
HIS 18
LYS 7
THR 19
VAL 20
GLU 21
LYS 27
D
GLY 188
ASH 392
TRP 246
SER 186
TYR 436
ASP 244
ASH 243
SER 325
PRO 123
HIE 438
GLY 157
ASP 439
ARG 265
LEU 322
PRO 321
CYS 158
GLY 159
THR 263
PHE 425
LYS 160
SER 161
VAL 162
E
ASP 186
SER 361
SER 236
HIE 237
GLN 285
GLY 238
ALA 286
SER 287
PRO 357
ARG 355
TRP 354
SER 353
VAL 352
TYR 397
ARG 180
THR 181
F
MET 44
ASN 80
LEU 81
LYS 82
PHE 275
TYR 276
HIE 277
ARG 79
MET 39
ASP 40
LEU 223
LYS 224
ALA 227
ASP 228
G
GLY 321
GLU 335
TYR 334
ILE 333
PRO 332
THR 337
SER 338
ASN 407
THR 341
GLU 396
GLN 465
MET 403
THR 469
THR 467
PHE 399
LEU 401
GLN 361
H
ARG 143
PHE 142
LEU 117
ILE 82
ILE 83
ILE 120
THR 121
GLU 85
LEU 150
GLN 112
LEU 110
GLN 154
GLU 153

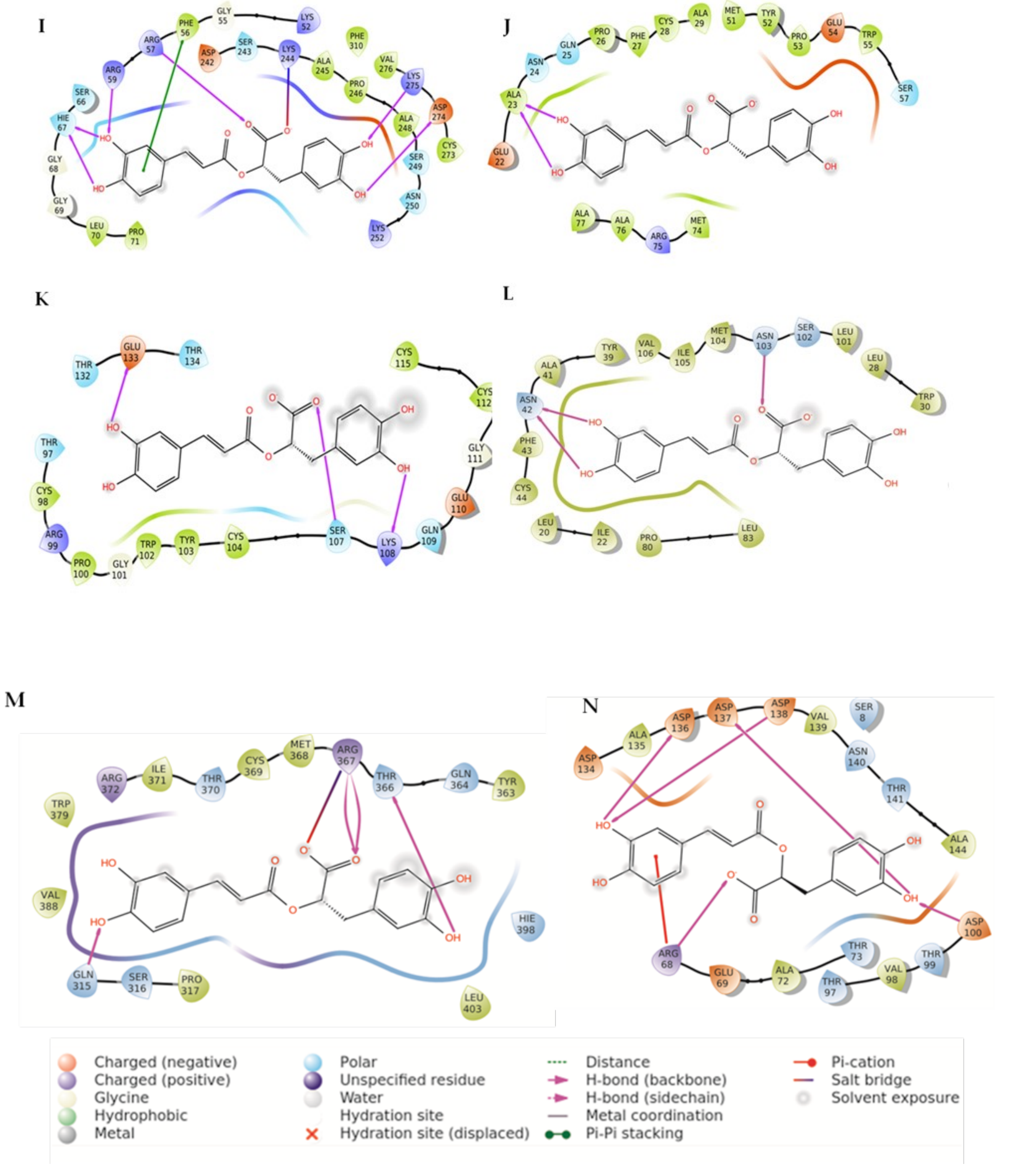

Supplementary Figure 1. Docking interactions of RA with Bad **(A)**, Bcl-2 **(B)**, cyt c **(C)**, Apaf1 **(D)**, caspase 9 **(E)**, caspase 3 **(F)**, caspase 8 **(G)**, IκBα **(H)**, NF-κB **(I)**, PKC-δ **(J)**, TNFR2 **(K)**, TGF-β1, **(L)**, SMAD3 **(M)**, and E-cadherin **(N)**. Selective receptors/proteins were retrieved from the publicly available Protein Data Bank (PDB).

Effect on the expressions of MAPKs and iNOS in vitro and in vivo

In in vitro assay, $CdCl_2$ (40 μM) caused significant activation of p38 and JNK MAPK signaling, which was visualized from significant ($p < 0.01$) up-regulation in the expressions of phospho-p38 and phospho-JNK in $CdCl_2$-exposed murine renal cells (Supplementary Figure 2A). On the other hand,

incubation of mouse proximal tubular epithelial cells with RA (40 μM) 1 h prior to $CdCl_2$ (40 μM) treatment significantly ($p < 0.01$) reciprocated p38 and JNK activation in mouse renal cells (Supplementary Figure 2A). $CdCl_2$ (40 μM) treatment to mouse proximal tubular epithelial cells significantly activated ($p < 0.01$) p53 and iNOS expressions in mouse proximal tubular epithelial cells; however, incubation of mouse renal cells with RA (40 μM) 1 h prior to $CdCl_2$ (40 μM) treatment significantly attenuated ($p < 0.01$) p53 and iNOS expressions (Supplementary Figure 2A).

In this study, $CdCl_2$ (4 mg/kg) treatment for 2 weeks significantly activated p38 and JNK MAPK signaling in mouse kidneys, which was visualized from significant ($p < 0.01$) up-regulation in the expressions of phospho-p38 and phospho-JNK in the kidneys of $CdCl_2$-treated experimental mice (Supplementary Figure 2B). On the other hand, RA (4 mg/kg) treatment significantly reciprocated p38 and JNK activation in the kidneys of experimental mice (Supplementary Figure 2B). $CdCl_2$ (4 mg/kg) treatment further caused significant ($p < 0.01$) activation of p53 and iNOS expressions in the kidneys of experimental mice (Figure 11). On the other hand, RA (4 mg/kg) treatment significantly reduced p53 ($p < 0.05$) and iNOS ($p < 0.01$) expressions in the kidneys of experimental mice (Supplementary Figure 2B).

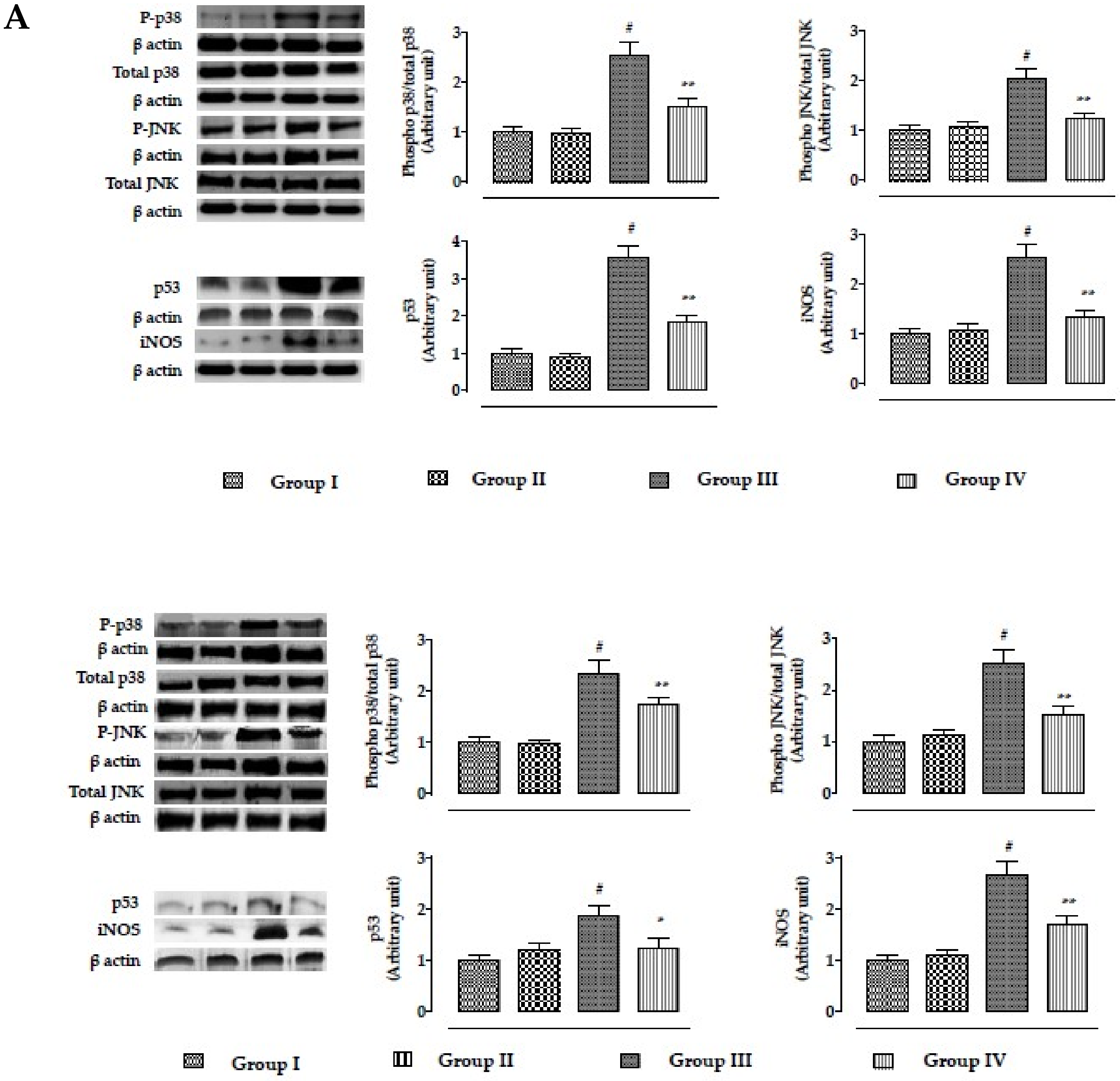

Supplementary Figure 2. The effects on MAPKs, and iNOS expressions in the absence ($CdCl_2$) and presence of RA ($CdCl_2$ + RA) in vitro (**A**) and in vivo (**B**). The relative band intensities were assessed

and the intensity of normal control band was allotted 1. β-actin served as loading control. Data were represented as mean ± SD, n = 3, (in vitro) or n = 6 (in vivo). #Values significantly ($p < 0.01$) differ from normal control. *Values significantly ($p < 0.05$) differ from $CdCl_2$ control. **Values significantly ($p < 0.01$) differ from $CdCl_2$ control.
